# Practical Synthesis of Antimicrobial Long Linear Polyamine Succinamides

**DOI:** 10.1021/acsbiomedchemau.2c00033

**Published:** 2022-10-11

**Authors:** Abdulaziz H. Alkhzem, Shuxian Li, Toska Wonfor, Timothy J. Woodman, Maisem Laabei, Ian S. Blagbrough

**Affiliations:** † Department of Pharmacy and Pharmacology, University of Bath, Bath BA2 7AY, U.K.; ‡ Department of Biology and Biochemistry, University of Bath, Bath BA2 7AY, U.K.

**Keywords:** antibacterial action, biofilm, hemolytic assays, linear polyamines, MRSA, *Pseudomonas aeruginosa*, spermine

## Abstract

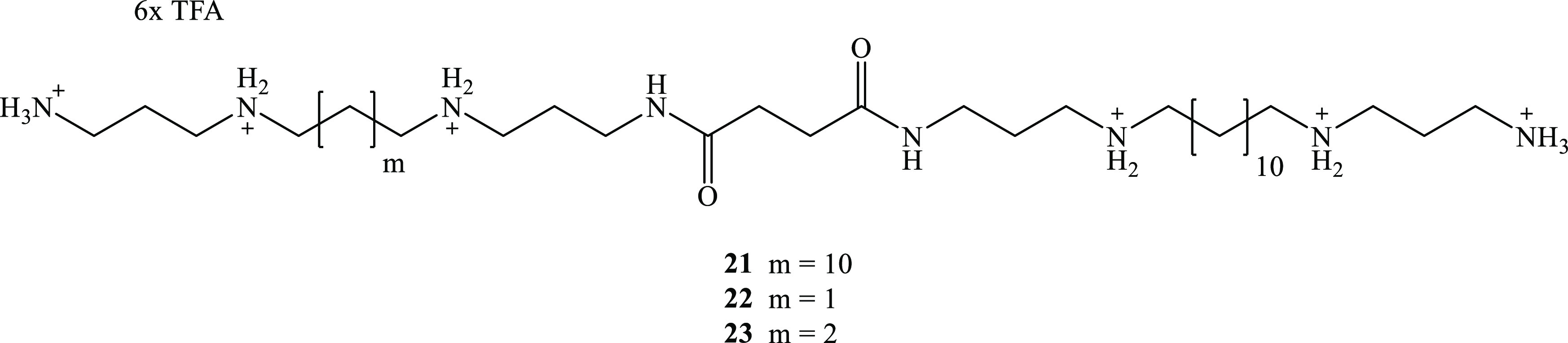

There are many severe bacterial infections notorious for their ability to become resistant to clinically relevant antibiotics. Indeed, antibiotic resistance is a growing threat to human health, further exacerbated by the lack of new antibiotics. We now describe the practical synthesis of a series of substituted long linear polyamines that produce rapid antibacterial activity against both Gram-positive and Gram-negative bacteria, including meticillin-resistant *Staphylococcus aureus*. These compounds also reduce biofilm formation in *Pseudomonas aeruginosa*. The most potent analogues are thermine, spermine, and 1,12-diaminododecane homo- and heterodimeric polyamine succinic acid amides. They are of the order of activity of the aminoglycoside antibiotics kanamycin and tobramycin as positive controls. Their low human cell toxicity is demonstrated in ex vivo hemolytic assays where they did not produce even 5% hemolysis of human erythrocytes. These long, linear polyamines are a new class of broad-spectrum antibacterials active against drug-resistant pathogens.

## Introduction

Antimicrobial resistance (AMR) is one of the top 10 current global health threats.^1,2^ The continuous increase of AMR among major bacterial pathogens is expected to result in 10 million deaths per year by 2050 with an estimated US$1 trillion in health-care costs globally.^3,4^ Estimates have indicated that up to US$100 trillion could be lost to the global economy due to decreased productivity.^3^ Despite the urgent need for investments in research and development of antibiotics, only two new antibiotic classes (oxazolidinones and lipopeptides) have emerged and been approved for clinical use.^5^ As a response to the lack of antibiotics in the clinical pipeline and the growing spread of antibiotic resistance, the WHO have generated a priority list to inform and direct research to tackle specific antibiotic-resistant human bacterial pathogens through the development of new and effective antibiotics.^6^ Accordingly, we have examined the activity of novel linear polyamines against seven major human bacterial pathogens identified on this list (*Enterococcus faecalis*, *E. faecium*, *Staphylococcus aureus*, *Klebsiella pneumoniae*, *Acinetobacter baumannii*, *Pseudomonas aeruginosa*, and *Escherichia coli*) and one emerging pathogen (*S. epidermidis*).

The positive charges of the amino groups along these linear polyamines^7^ and the elongated polyamine amides are potentially a key factor in their rapid antimicrobial activity against both Gram-positive and Gram-negative bacteria including meticillin-resistant *S. aureus* (MRSA), potentially primarily acting by depolarization of the cytoplasmic membrane and permeabilization of the bacterial outer membrane.^8,9^ Linking two of the same or different polyamines via amide bonds can be achieved by introducing a carboxylic acid group on the first polyamine, then coupling to a free primary amine in the second polyamine. If the addition of positive charges^10−12^ increases the antimicrobial activity of linear polyamines,^13−17^ synthesizing homo- or heterodimeric polyamines will increase the total net charge compared to their monomeric counterparts.^18^

## Results and Discussion

### Synthesis of 1,20-Diamino-4,17-diazaicosane (3.12.3) 3 and Selective Protection of the Reactive Amines on Polyamines

A two-step procedure was used to form tetraamine **3** which incorporates the *N*^1^,*N*^12^-bis(3-aminopropyl) pattern of substituents on 1,12-diaminododecane.^19,20^ Using the trivial nomenclature for linear polyamines of the (poly)methylene count, where tetraamine thermine **4** is indicated as 3.3.3 and spermine **5** is known as 3.4.3, this long-chain target tetraamine is accurately described as 3.12.3. Commercially available 1,12-diaminododecane **1** was reacted with two equivalents of acrylonitrile in EtOH at 20 °C to undergo two 1,4-Michael addition reactions to obtain dinitrile **2**(20) in 87% yield (see Figures S1 and S2). The formation of some tri-Michael adducts was observed. ^13^C NMR spectroscopy showed a nitrile carbon signal of low intensity at 118.8 ppm. Using LiAlH_4_ in anhydrous THF was investigated to reduce the nitrile functional groups to primary amines, but this gave a complex mixture.^21^ Pd/C (10%) was investigated as a catalyst under hydrogen gas with little success,^22^ likewise using Raney nickel as a catalyst.^23^ However, the nitrile functional groups were successfully reduced to primary amines using catalytic Raney nickel and sodium hydroxide (co-catalyst) under a hydrogen pressure of 1 bar to afford tetraamine **3** in 75% yield (see Figure S3).^24,25^

**Figure 1:**
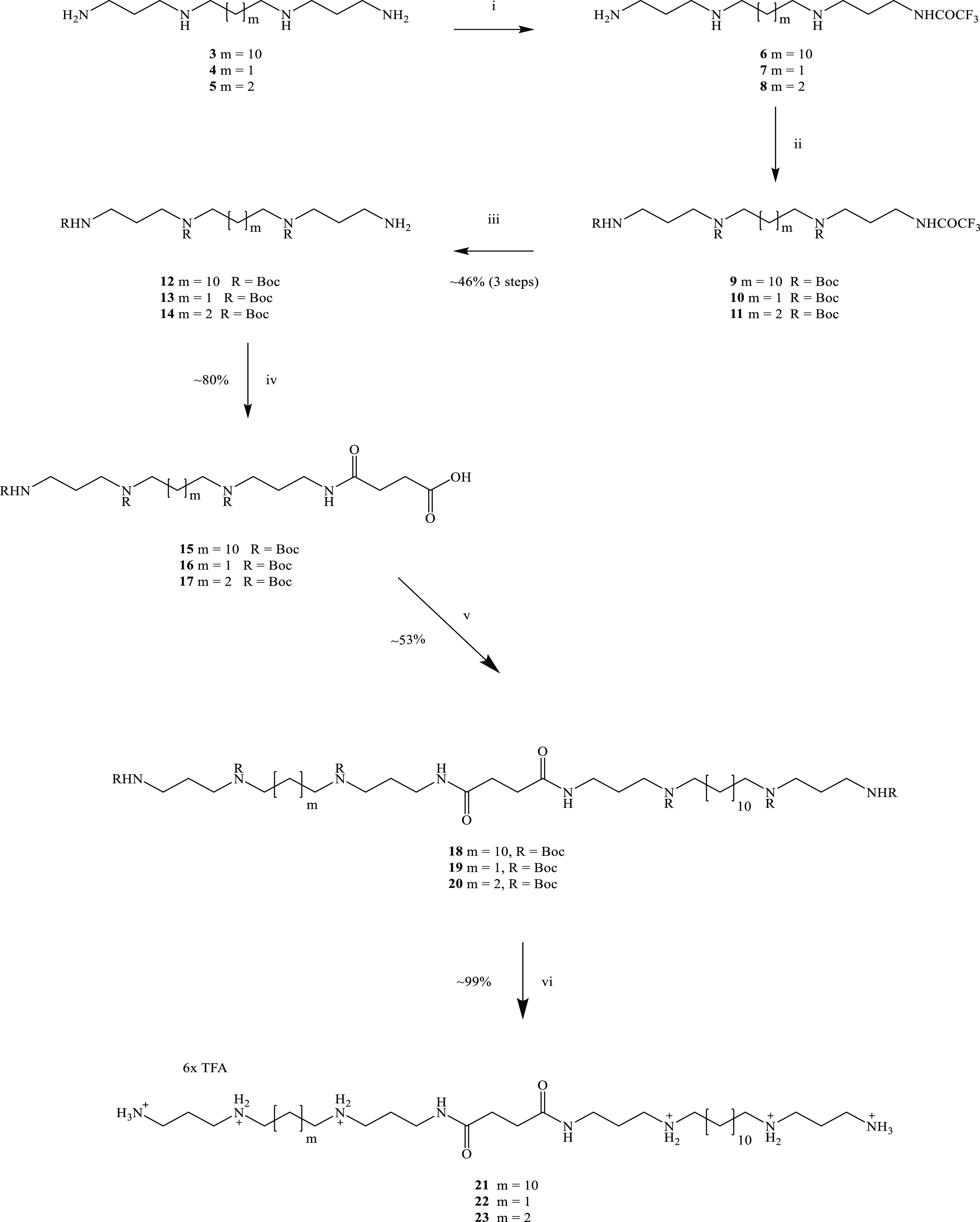
Reagents and conditions: (i) EtOOCCF_3_, MeOH, −78 °C, 18 h; (ii) Boc_2_O, MeOH, 0–20 °C, 18 h; (iii) aq. NH_3_, 20 °C, 18 h; (iv) succinic anhydride, anhydrous pyridine, 20 °C, 18 h; (v) HBTu, R–NH_2_, TEA, anhydrous DMF, 20 °C, 18 h; and (vi) DCM/TFA (9:1 v/v), 20 °C, 18 h.

The ability to covalently link polyamines requires selective protection, in order to avoid unwanted side products. However, the protection of three amines out of four is always low yielding and needs substantial chromatographic purification. Mono-protection using benzyl chloroformate (CbzCl) or di-*tert*-butyldicarbonate (Boc anhydride) is not practical due to low yields and requires more substantial chromatographic purification. Geall and Blagbrough reported that using trifluoroacetyl as a protecting group for one amine out of four can be controlled by decreasing the temperature and the concentration.^26^ Subsequent removal under basic conditions makes trifluoroacetyl an ideal protecting group compared to Cbz and Boc for the purpose of gram-scale protection of polyamines in preparing unsymmetrical polyamine amides. The ratio between −NH_2_ and ethyl trifluoroacetate (the source of the protecting group) is important not only to avoid protection of both primary amines but also to avoid protection of the more sterically hindered secondary amines on **3**, **4**, and **5**. Thus, using this approach, via mono-trifluoroacetamides **6**, **7**, and **8**, protection of the three remaining amino groups in the fully protected derivatives **9**, **10**, and **11**, respectively, the corresponding three tri-Boc protected derivatives **12**, **13**, and **14** were obtained following trifluoroacetyl removal, Figure 1.

### Synthesis of Polyamine Amide-Succinic Half Acids and Their Conversion into Homo- and Heterodimeric Linear Polyamine Succinamides

Succinic anhydride was selected to link two of the same or different linear polyamines, introducing a short (4 carbon) spacer and a useful carboxylic acid functional group from which amides may be obtained, for example, from the same or different long linear polyamines incorporating a *N*^1^,*N*^12^-bis(3-aminopropyl)-1,12-diaminododecane moiety.^27^ Compounds **15**, **16**, and **17** were synthesized by the addition of one equivalent of succinic anhydride to a solution of each tri-Boc protected polyamine **12**, **13**, and **14** in anhydrous pyridine at 20 °C, Figure 1. All spectral data confirmed that the reactions successfully occurred.

The ^1^H-^13^C HMBC NMR spectrum of succinic amide acid **17**, the tri-Boc compound derived from spermine (3.4.3, **5**) shows that there are two triplets at 2.45 and 2.58 ppm in the ^1^H NMR spectrum (Figure S5), which represent the two CH_2_ groups between the amide group and the carboxylic acid, and carbon signals at 173.4 and 174.8 ppm in the ^13^C NMR spectrum, which represent the carbonyl carbons of the carboxylic acid and the amide group. ^1^H-^13^C cross-peaks in the HMBC NMR spectra between the two triplets and the two carbon signals were observed, see Figure S4. Compounds **15**, **16**, and **17** were separately coupled to one equivalent of the primary amine **12** to obtain the corresponding target homo- and heterodimeric protected polyamines **18**, **19**, and **20** in good yield by the addition of one equivalent of HBTu to activate the carboxylic acid in anhydrous DMF at 20 °C. ^1^H NMR spectra show a singlet at 2.52 ppm integrating for the four protons between the two new amide groups (RNHCO**CH**_**2**_**CH**_**2**_CONHR) rather than a triplet, as observed for succinic amide half acids **15**, **16**, and **17**, Figure S5. Also, there is one carbonyl signal observed at ∼175 ppm with higher intensity, from both the amide carbonyl groups, and the disappearance of the carboxylic acid signal in the ^13^C NMR spectrum. Deprotection of the Boc protecting groups in compounds **18**, **19**, and **20** proceeded smoothly using TFA/DCM (1:9 v/v) stirred for 18 h, then concentrated in vacuo, and lyophilized to yield desired heterodimeric linear polyamines **21**, **22**, and **23** as poly-TFA salts confirmed by MS, IR, and NMR spectroscopy, see Figures 1 and S6. For unambiguous assignment, all the protons and carbons of the three target compounds **21**, **22**, and **23** were assigned using 1D (Figure S6), 2D, ^1^H-^13^C HSQC, and ^1^H-^13^C HMBC NMR spectroscopy (Figures S7–S15) where the detail in these unambiguous spectroscopic assignments is set out.

### Microbiological Activity of Homo- (21) and Heterodimeric (**22**, and **23**) Linear Polyamines Incorporating a *N*^1^,*N*^12^-Bis(3-aminopropyl)-1,12-diaminododecane (3.12.3) Moiety

The antibacterial activity was measured for compounds **21**, **22**, and **23** on different bacterial strains (Table 1). In particular, activity was found against *P. aeruginosa* PAO1 and *S. aureus* SH1000, at levels comparable to those of the positive controls, kanamycin (MIC = 32 μg/mL) and tobramycin (MIC = 1–2 μg/mL). In order to investigate if there was any significant difference caused by the counterions, homo-dimer **21** was prepared not only as its poly-(hexa)-TFA salt but also as its poly-HCl salt, by Boc-protecting group removal with 4 M HCl in 1,4-dioxane at 20 °C. The solution was stirred for 18 h, then concentrated in vacuo, and lyophilized to yield the desired product as a white powder. The poly-HCl salt of homo-dimer **21** was as equally potent as its hexa-TFA salt against *S. aureus* SH1000, MIC = 4 μg/mL. Also, there were good antibiofilm data for all three polyamine succinamides, with 8–32 μg/mL MIBC antibiofilm levels against PAO1 and 4–64 μg/mL MIBC against SH1000 (Table 2). For toxicity quantified by measurement on human erythrocytes (hRBCs), even at concentrations of 512 μg/mL, there was less than 5% hemolysis of hRBCs, shown in the hemolysis HC5 concentration data for compounds **21**, **22**, and **23** (Table 3) where hemolysis HC5 is the concentration required to induce hemolysis of 5% of the erythrocyte population.

**Table 1:** Microbiological Activity of Compounds **21**, **22**, and **23** on Different Bacterial Strains

		minimum inhibitory concentration (MIC) (μg/mL)					
bacterial species	gram stain	spermidine 3.4	kanamycin	tobramycin	22 AHA1268	23 AHA1282	21 AHA1394
*E. faecalis*	+ve	>128	>128	>128	>128	>128	>128
*E. faecium*	+ve	>128	64	32	>128	>128	64
*S. epidermidisRP62A*	+ve	>128	>128	64	64	64	4
*K. pneumonia*	–ve	>128	4	16	128	128	8–16
*A. baumannii*	–ve	>128	2	2	>128	>128	32–64
*E. coliK12*	–ve	>128	16	32	>128	>128	16–32
*P. aeruginosaPAO1*	–ve	>128	32	1–2	32	16	4
*S. aureusSH1000*	+ve	>128	2	0.25	32	32	4

**Table 2:** Minimum Inhibitory Biofilm Concentrations (MIBCs) of Compounds **21**, **22**, and **23**

	MIBC biofilm (μg/mL)	MIBC biofilm (μg/mL)
compound	SH1000 (MSSA)	PAO1
**21**	4–8	8
**22**	64	8–32
**23**	64	8–32

**Table 3:** Hemolysis (HC5) Concentrations of Compounds **21**, **22**, and **23**

compound	hemolysis (HC5) (μg/mL)
**21**	>512
**22**	>512
**23**	>512
kanamycin	>512
tobramycin	>512

Quaternary ammonium compounds (QACs) typically contain trimethylammonium or benzyldimethyl alkylammonium units where, as they are quaternized amines, the positive charge is permanent, typically with a corresponding chloride or bromide counterion. The three target long linear polyamines reported here are not QACs. They are neither quaternary nor permanently positively charged. QACs are widely applied as biocides in household and in numerous industrial products used for, for example, water treatment, antifungal treatment in horticulture, in pharmaceutical and everyday consumer products as preservatives, foam boosters, and detergents. Therefore, QACs occur in the aquatic and terrestrial environment all over the world. The excessive use of QACs has resulted in the emergence of antibiotic-resistant bacteria as QACs act as detergents targeting the lipid wall. Therefore, they do kill microbes working against a broad range of microorganisms, but still their modes of action are not finalized.^28^ As Hegstad and co-workers reviewed,^29^ the resistance toward QACs is widespread among a diverse range of microorganisms. This resistance occurs by several mechanisms, for example, modifications in the composition of the membrane, expression of efflux pump genes, and/or expression of stress response and repair systems. Development of resistance in both pathogenic and nonpathogenic bacteria has been related to application in human medicine and the food industry. QACs in cosmetic products inevitably come into intimate contact with the skin or mucosal linings in the mouth and thus they are likely to add to the selection pressure toward more QAC-resistant microorganisms among the skin or mouth flora. There is increasing evidence of co-resistance and cross-resistance between QACs and a range of other clinically important antibiotics and disinfectants. The use of QACs may have driven the fixation and spread of certain resistance cassette collectors (class 1 integrons), currently responsible for a major part of AMR in Gram-negative bacteria. More indiscriminate use of QACs such as in cosmetic products may drive the selection of further new genetic elements that will aid in the persistence and spread of AMR and thus in further limiting our few remaining treatment options for microbial infections.^29^

Woster and co-workers have reported^9^ a series of substituted diamines that produce rapid bactericidal activity against both Gram-positive and Gram-negative bacteria. The mode of action of these antibacterial diamines is by targeting bacterial membranes. The linear diamines are based on a dithioacylated 3.5.3 polyamine skeleton, terminating in lipophilic phenyl thioureas. They also reduce biofilm formation and promote biofilm dispersal in *P. aeruginosa*. These exciting new compounds typically display MIC of 2 μg/mL (against MRSA) and 8 μg/mL (against *P. aeruginosa*). These linear diamines act primarily by rapid depolarization of the cytoplasmic membrane and permeabilization of the bacterial outer membrane. Also significant are the results in human cell toxicity assays, where they showed limited adverse effects leading Woster and colleagues to conclude that such linear polyamine-derived diamines represent a new class of broad-spectrum antibacterials against drug-resistant pathogens.^9^

Haldar and co-workers reported^30^ di-Phe-derived lipophilic triamides of norspermidine (3.3) are membrane-active with excellent selective antibacterial activity against various wild-type bacteria (Gram-positive and Gram-negative) and drug-resistant bacteria. These are diamines from the two aromatic amino acids (Phe). Such rapidly bactericidal natural and synthetic membrane-active antibacterial agents offer hope as potential solutions to the problem of bacterial resistance as their membrane-active nature imparts a low propensity for the development of resistance and they have potential as therapeutic agents to tackle multidrug-resistant bacterial infections. In related studies, Konai and Haldar reported^31^ membrane-active lipophilic norspermidine-derived triamides that are di-Lys conjugates and therefore they typically carry four positive charges. As we have shown herein, the toxicity evaluated against the lysis of hRBCs showed that such linear tetraamines did not cause significant hemolysis. To demonstrate that they are effective as selective antibacterial agents, the hemolytic activity assay for the common antiseptics, benzalkonium chloride (C12 from a range of C8–C18, BAC-12) and didecyl dimethyl ammonium bromide (DDAB-10), and other cationic amphiphiles, QACs such as 1-dodecyl trimethyl ammonium bromide (DTAB) and 1-hexadecyl trimethyl ammonium bromide (C16 is cetyl, CTAB), were also performed. The QACs are highly toxic, resulting in hRBC lysis at very low concentrations (27–218 μg/mL),^31^ whereas our compounds are antibacterial at very low concentrations and remain nontoxic even at significantly higher concentrations of >512 μg/mL. Such compounds are selective antibacterial agents as they do not display toxicity against mammalian HeLa cells, determined using the MTT assay and found to be >70 μg/mL which is manyfold higher compared to the concentration required for bactericidal activity.^31^

## Conclusions

The practical synthesis of three long linear polyamine amides that produce rapid antibacterial activity against both Gram-positive and Gram-negative bacteria, including MRSA, is described. All these compounds also effectively reduce biofilm formation in *P. aeruginosa*. These potent analogues are thermine, spermine, and bis-*N*^1^,*N*^12^(3-aminopropyl)-1,12-diaminododecane homo- and heterodimeric polyamine succinic acid amides with activity of the order of the aminoglycoside antibiotics kanamycin and tobramycin as positive controls. These data are important because they reveal that long, linear polyamines are a new chemical class of broad-spectrum antibacterial agents active against drug-resistant pathogens. Such polyamines cause a low (<5%) incidence of hRBC hemolytic toxicity. These data show and confirm that some linear polyamine analogues possess antibacterial activity against both Gram-positive and Gram-negative bacterial strains.

## Experimental Section

### Materials and General Methods

D_2_O, CD_3_OD, and CDCl_3_ were purchased from Goss Scientific (UK). Aqueous ammonia (32%), dichloromethane (DCM), dimethylformamide (DMF), ethanol (EtOH), ethyl acetate, methanol (MeOH), and triethylamine (TEA) were purchased from VWR (UK). Anhydrous pyridine, anhydrous dimethylformamide (DMF), and chloroform were purchased from Fisher Scientific (UK). Acrylonitrile, 1,12-diaminododecane, di-*tert*-butyl dicarbonate [(Boc)_2_O], ethyl trifluoroacetate, ninhydrin, norspermine (thermine), Raney-Nickel, spermidine, spermine, sodium hydroxide (NaOH), succinic anhydride, *N*,*N*,*N*′,*N*′-tetramethyl-*O*-(1*H*-benzotriazol-1-yl)uronium hexafluorophosphate (HBTu), trifluoroacetic acid (TFA), potassium bromide (KBr), and sodium sulfate (Na_2_SO_4_) were purchased from Sigma-Aldrich (UK).

Column chromatography was performed over silica gel 60–120 mesh (purchased from Sigma-Aldrich, UK) using different ratios of MeOH, EtOH, ethyl acetate, DCM, and aqueous ammonia (32%) as eluents. Thin-layer chromatography (TLC) was performed over silica gel using aluminum-backed sheets coated with Kieselgel 60 F_254_ purchased from Merck (UK). Ninhydrin TLC spray reagent was used for detecting amine functional groups [ninhydrin (0.2 g) in 100 mL EtOH].

### Bacterial Strains, Culture Conditions, Minimum Inhibitory Concentration Determination

Bacterial strains used in this study are listed in Table 1. *S. aureus, S. epidermidis, E. faecalis, and E. faecium* were grown on tryptic soy agar (TSA; Sigma-Aldrich, UK) for 18 h at 37 °C. *K. pneumoniae, A. baumannii, P. aeruginosa, and E. coli* were grown on Luria–Bertani agar (LBA; Sigma-Aldrich, UK) for 18 h at 37 °C. The minimum inhibitory concentration of polyamine compounds against the above bacterial species was determined using the broth microdilution method as described by the Clinical and Laboratory Standards Institute (CLSI).^32^

Individual pure colonies of the above bacterial species were used to inoculate separate 15 mL polystyrene test tubes (Thermo Fisher) containing 3 mL of cation-adjusted Mueller–Hinton broth (MHB; Oxoid). Growth agar and broth were made, according to manufacturer’s instructions. Bacterial cultures were incubated for 18 h at 37 °C with shaking at 180 rpm (New Brunswick Innova 44/R incubator). The 18 h bacterial cultures were subsequently diluted 1:100 in fresh MHB and cultured at 37 °C with shaking at 180 rpm to an exponential phase of growth, defined as reaching an absorbance (OD_600nm_) within the range of 0.5–0.6. Absorbance was measured using a 1 mm cuvette and DS-11 spectrophotometer (DeNovix). Polyamine compounds were reconstituted in sterile deionized water. Compounds were diluted in MHB and dispensed into a 96-well round bottom microtiter plate (Costar) to a final concentration range of 128–2 μg/mL. Aliquots of 0.5 McFarland standardized inoculum of bacteria were dispensed into wells containing polyamine compounds to a final inoculum of 5 × 10^5^ CFU/mL. Bacterial cultures were grown statically at 37 °C for 24 h (Thermo Scientific Heratherm). The MIC was defined as the lowest concentration of compound to result in no visible growth measured through inspection of turbidity. Tobramycin sulfate and kanamycin sulfate were used as antibiotic (positive) controls.

### Ex Vivo Hemolytic Assay

Informed consent was gained from three healthy donors prior to blood donation. Experiments were approved by the University of Bath, Research Ethics Approval Committee for Health (REACH) [reference: EP 18/19 108]. Whole blood (10 mL) was obtained from three healthy donors, drawn directly into K_2_-EDTA-coated Vacutainer tubes (BD Biosciences) to prevent coagulation. Tubes were centrifuged at 500*g* for 10 min at 4 °C (Eppendorf 5810R), the plasma layer was aspirated, discarded, and the remaining hematocrit was filled to the original volume with sterile saline solution (150 mM NaCl) and gently mixed by inversion. Blood cells were washed three times according to this above procedure, then after the final wash, hematocrit was resuspended in sterile phosphate buffered saline (PBS; Oxoid) to the original volume. Hematocrit was diluted to 2% (v/v) in PBS. Blood cells were aliquoted (100 μL) in triplicate in a 96-well microtiter plate. Equal volumes of polyamine compound diluted in PBS were added to a final concentration of 512–16 μg/mL and incubated for 1 h at 37 °C. Blood cells incubated with saline served as a measure of spontaneous lysis of erythrocytes and therefore as a negative control buffer. Total (hemo)lysis of erythrocytes was obtained following incubation with Triton X-100 (Sigma-Aldrich, UK) (2% v/v) as a positive control. Following incubation, plates were centrifuged at 500*g* for 5 min and 100 μL of supernatant was transferred to a new 96-well plate and the absorbance (OD_404nm_) was measured using a Sunrise absorbance microplate reader (Tecan Life Sciences). Degree of hemolysis was expressed as % hemolysis (see equation) relative to spontaneous lysis controls
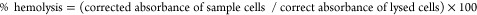


Each experiment was performed using three technical repeats, and values were derived from three biological replicates from three different blood donors.

### Instrumentation

NMR spectra including ^1^H, ^13^C, HSQC, HMBC, were recorded on Bruker Avance III (operating at 500.13 MHz for ^1^H and 125.77 MHz for ^13^C) spectrometers at 25 °C. MestReNova has been used for processing the spectra. ^1^H and ^13^C chemical shifts (δ) were observed and are reported in parts per million (ppm) relative to tetramethylsilane at 0.00 ppm as an internal reference or to the residual solvent peak, HDO at 4.79 ppm. High resolution time-of flight mass spectra were obtained on a Bruker Daltonics “micrOTOF” mass spectrometer using electrospray ionization (ESI) (loop injection +ve ion mode). PerkinElmer 65 spectrum FT-IR spectroscopy was used to obtain the IR spectra. Anhydrous potassium bromide (KBr) discs were prepared for solid samples.

### General Procedure A: Boc Removal

A solution of fully Boc polyamine in DCM (9 mL) was deprotected by adding TFA (1 mL) at 20 °C. The solution was stirred for a further 18 h, then concentrated in vacuo and lyophilized to yield the desired product as a pale yellow oil (poly-TFA salt).

#### *N*^1^,*N*^12^-Bis(2-cyanoethyl)-1,12-diaminododecane 2

A solution of 1,12-diaminododecane **1** (1.00 g, 4.99 mmol) in EtOH (20 mL) under nitrogen was treated with acrylonitrile (0.53 g, 9.98 mmol, 2 equiv) at 20 °C and stirred for a further 18 h, then concentrated in vacuo, and the crude material was purified over silica gel, (DCM/MeOH; 9.5:0.5 to 9:1 v/v). After combining fractions and concentrating them, the desired product **2** was obtained as a white solid (1.33 g, 87%), homogeneous by TLC analysis (*R*_f_ = 0.4, DCM/MeOH, 9:1 v/v). HRMS: found 307.2792 (*m*/*z*), C_18_H_35_N_4_ requires 307.2856 (*m*/*z*) [M + H]^+^; IR (KBr disc); 2253 s (CN) cm^–1^ see Figure S2. ^1^H NMR, 500 MHz (CDCl_3_): 1.25–1.34 (m, 16H, 7-CH_2_, 8-CH_2_, 9-CH_2_, 10-CH_2_), 1.45–1.55 (m, 4H, 6-CH_2_), 2.52 (t, *J* = 6.6 Hz, 4H, 2-CH_2_), 2.62 (t, *J* = 6.6 Hz, 4H, 5-CH_2_), 2.93 (t, *J* = 6.6 Hz, 4H, 3-CH_2_); ^13^C NMR, 125.77 MHz (CDCl_3_): 18.68 (2-CH_2_), 27.0, 29.5, 29.5, 29.5, 30.0 (6-CH_2_, 7-CH_2_, 8-CH_2_, 9-CH_2_, 10-CH_2_), 45.0 (3-CH_2_), 49.2 (5-CH_2_), 118.8 (2× CN) in agreement with those spectral data previously reported.^15^ MS data also showed the correct mass for tri-1,4-Michael adducts: found 360.3066, C_21_H_38_N_5_ requires 360.3049 [M + H]^+^.

#### *N*^1^,*N*^12^-Bis(3-aminopropyl)-1,12-diaminododecane 3

A solution of bis(2-cyanoethyl) **2** (2.00 g, 6.52 mmol) and NaOH (0.78 g, 19.6 mmol, 3 equiv) was dissolved in EtOH (30 mL). Raney nickel (∼0.5 g) was added to the mixture. The atmosphere over the solution was evacuated and replaced with N_2_ gas three times and then replaced with H_2_. The solution was stirred under H_2_ for a further 18 h at 20 °C, filtered through Celite with EtOH and then concentrated in vacuo. The crude material was extracted with a mixture of chloroform/MeOH (85:15 v/v, 10× 15 mL). The combined organic extracts were dried (Na_2_SO_4_), filtered, and concentrated in vacuo to yield the desired tetraamine **3**, 1,20-diamino-4,17-diazaicosane, as a pale yellow oil (1.55 g, 75%), homogeneous by TLC analysis (*R*_f_ = 0.3 (EtOAc/DCM/ethanol/aq. ammonia (32%), 3:3:3:1 v/v/v). HRMS: found 315.3581 (*m*/*z*), C_18_H_43_N_4_ requires 315.3482 (*m*/*z*) [M + H]^+^; IR (film): 3271–3396 (NH, NH_2_), and 2932 (CH_2_) cm^–1^; ^1^H NMR, 500 MHz (CDCl_3_): 1.24–1.30 (m, 16H, 7-CH_2_, 8-CH_2_, 9-CH_2_, 10-CH_2_), 1.45–1.50 (m, 4H, 6-CH_2_), 1.64 (m, 4H, 2-CH_2_), 2.59 (t, *J* = 7.1 Hz, 4H, 5-CH_2_), 2.66 (t, *J* = 7.1 Hz, 4H, 3-CH_2_), 2.76 (t, *J* = 7.1 Hz, 4H, 1-CH_2_); ^13^C NMR, 125.77 MHz (CDCl_3_): 27.3, 29.5, 30.1 (6-CH_2_, 7-CH_2_, 8-CH_2_, 9-CH_2_, 10-CH_2_), 33.9 (2-CH_2_), 40.5 (1-CH_2_), 47.9 (3-CH_2_), 50.2 (5-CH_2_), Figure S3 in agreement with those data previously reported.^33^

Tri-Boc **12**, **13**, and **14** were synthesized by the addition of one equivalent of ethyl trifluoroacetate to a methanolic solution of starting materials **3**, **4**, and **5** at −78 °C to obtain mono-trifluoroacetamides **6**, **7**, and **8**. HRMS: found 411.3241, C_20_H_42_F_3_N_4_O requires 411.3232 [M + H]^+^ for compound **6**. HRMS: found 285.1810, C_11_H_24_F_3_N_4_O requires 285.1824 [M + H]^+^ for compound **7**. HRMS: found 299.1971, C_12_H_26_F_3_N_4_O requires 299.1980 [M + H]^+^ for compound **8**. The products were not isolated. In the same methanolic solution, three equivalents of di-*tert*-butyldicarbonate were added to protect fully polyamines **9**, **10**, and **11**. HRMS: found 733.4791, C_35_H_65_NaF_3_N_4_O_7_ requires 733.4805 [M + Na]^+^ for compound **9**. HRMS: found 585.3411, C_26_H_48_F_3_N_4_O_7_ requires 585.3397 [M + H]^+^ for compound **10**. HRMS: found 621.3546, C_27_H_49_NaF_3_N_4_O_7_ requires 621.3553 [M + Na]^+^ for compound **11**. The trifluoroacetamide group was selectively deprotected by increasing the pH to above 11 with conc. aq. ammonia to afford polyamines **12**, **13**, and **14**, each substituted with one unmasked amino group. The spectral data obtained for compounds **13** and **14** agreed with those reported previously.^26^

#### (*N*^1^,*N*^4^,*N*^17^-Tri-*tert*-butoxycarbonyl)-1,20-diamino-4,17-diazaicosane 12

A solution of tetraamine **3** (1 g, 3.18 mmol) in MeOH (150 mL), at −78 °C under nitrogen, was treated with ethyl trifluoroacetate (0.45 g, 3.18 mmol, 1 equiv) added dropwise over 15 min. Stirring was continued for a further 45 min, then the temperature was increased to 20 °C for 18 h to afford the mono-trifluoroacetamide **6**. Without purification, the remaining amino functional groups were protected with an excess of di-*tert*-butyldicarbonate (2.78 g, 12.7 mmol, 4.0 equiv) in MeOH (20 mL) at 0 °C over 10 min. The reaction mixture was then warmed to 20 °C and stirred for a further 18 h to afford the fully protected polyamine **9**. The trifluoroacetate protecting group was then removed by increasing the pH of the solution to above 11 with conc. aq. ammonia (32%) and then stirring at 20 °C for 18 h. The solution was concentrated under reduced pressure and using column chromatography was eluted with DCM in MeOH (9.5:0.5 v/v). After combining fractions and concentrating them, the desired product **12** was obtained as a pale yellow oil (0.89 g, 45%), homogeneous by TLC analysis [*R*_f_ = 0.6, DCM/MeOH/aq. ammonia (32%), 70:10:1 v/v/v]. HRMS: found 615.4982 (*m*/*z*), C_33_H_67_N_4_O_6_ requires 615.4979 (*m*/*z*) [M + H]^+^; IR (KBr disc); 1677 (C=O) cm^–1^; ^1^H NMR, 500 MHz (CDCl_3_): 1.22–1.31 (m, 16H, 7-CH_2_, 8-CH_2_, 9-CH_2_, 10-CH_2_, 11-CH_2_, 12-CH_2_, 13-CH_2_, 14-CH_2_, overlapping), 1.41–1.53 (m, 31H, 9× CH_3_, Boc, 6-CH_2_, 15-CH_2_, overlapping), 1.59–1.68 (m, 2H, 2-CH_2_), 1.86–1.97 (m, 2H, 19-CH_2_), 2.90–2.99 (t, *J* = 6.4 Hz, 2H, 1-CH_2_), 3.04–3.36 (m, 10H, 3-CH_2_, 5-CH_2_, 16-CH_2_, 18-CH_2_, 20-CH_2_); ^13^C NMR, 125.77 MHz (CDCl_3_): 25.3 (19-CH_2_), 26.7, 26.9, 29.3, 29.5 (7-CH_2_, 8-CH_2_, 9-CH_2_, 10-CH_2_, 11-CH_2_, 12-CH_2_, 13-CH_2_, 14-CH_2_, overlapping), 28.7, 28.4 (9× CH_3_, Boc, 2-CH_2_, 6-CH_2_, 15-CH_2_, overlapping), 36.3 (1-CH_2_), 42.7, 44.0, 46.8, 47.0, 47.3 (3-CH_2_, 5-CH_2_, 16-CH_2_, 18-CH_2_, 20-CH_2_, overlapping), 79.8–81.2 (3× Cq, Boc), 155.7–157.5 (3× C=O, Boc).

#### (*N*^1^,*N*^4^,*N*^8^-Tri-*tert*-butoxycarbonyl)-1,11-diamino-4,8-diazaundecane 13

A solution of norspermine (thermine, 3.3.3) **4** (2.00 g, 10.6 mmol) in MeOH (150 mL), at −78 °C under nitrogen, was treated with ethyl trifluoroacetate (1.51 g, 10.6 mmol, 1 equiv) dropwise over 15 min. Stirring was continued for a further 45 min, then the temperature was increased to 20 °C for 18 h to afford the mono-trifluoroacetamide **7**. Without purification, the remaining amino functional groups were protected with an excess of di-*tert*-butyldicarbonate (6.95 g, 31.8 mmol, 3.0 equiv) in MeOH (20 mL) at 0 °C over 10 min. The reaction mixture was then warmed to 20 °C and stirred for a further 18 h to afford the fully protected polyamine **10**. The trifluoroacetate protecting group was then removed by increasing the pH of the solution to above 11 with conc. aq. ammonia (32%) and then stirring at 20 °C for 18 h. The solution was concentrated under reduced pressure. The crude product was purified via column chromatography with DCM in MeOH (9.5:0.5 v/v). After combining fractions and concentrating them, the desired product **13** was obtained as a colorless oil (2.50 g, 48%), homogeneous by TLC analysis (*R*_f_ = 0.5, DCM/MeOH/aq. ammonia (32%), 70:10:1 v/v/v). HRMS: found 489.3641 (*m*/*z*), C_24_H_49_N_4_O_6_ requires 489.3573 (*m*/*z*) [M + H]^+^; IR (film): 1689 (C=O) cm^–1^; ^1^H NMR, 500 MHz (CDCl_3_): 1.37–1.51 (m, 27H, 9× CH_3_, Boc), 1.66–1.78 (m, 4H, 2-CH_2_, 6-CH_2_, overlapping), 1.88–193 (m, 2H, 10-CH_2_), 2.91–2.98 (t, *J* = 6.4 Hz, 2H, 11-CH_2_), 3.05–3.38 (m, 10H, 1-CH_2_, 3-CH_2_, 5-CH_2_, 7-CH_2_, 9-CH_2_); ^13^C NMR, 125.77 MHz (CDCl_3_): 25.9 (10-CH_2_), 27.4, 27.8, 29.0 (9× CH_3_, Boc, 2-CH_2_, 6-CH_2_, overlapping), 36.5 (11-CH_2_), 40.6, 40.9, 46.1, 46.4 (1-CH_2_, 3-CH_2_, 5-CH_2_, 7-CH_2_, 9-CH_2_), 79.9–80.8 (3× Cq, Boc), 156.5–158.0 (3× C=O, Boc).

#### (*N*^1^,*N*^4^,*N*^9^-Tri-*tert*-butoxycarbonyl)-1,12-diamino-4,9-diazadodecane 14

A solution of spermine (3.4.3) **5** (0.50 g, 2.47 mmol) in MeOH (100 mL), at −78 °C under nitrogen, was treated with ethyl trifluoroacetate (0.35 g, 2.47 mmol, 1 equiv). The ethyl trifluoroacetate was added dropwise over 20 min, stirring was continued for a further 30 min, then the temperature was increased to 20 °C to afford predominantly the mono-trifluoroacetamide **8**. Without purification, the remaining amino functional groups were protected by an excess of di-*tert*-butyldicarbonate (1.61 g, 7.41 mmol, 3.0 equiv) in MeOH (15 mL) over 10 min. The reaction was then warmed to 20 °C and stirred for a further 18 h to afford the fully protected polyamine **11**. The trifluoroacetate protecting group was then removed by increasing the pH of the solution to above 11 with conc. aq. ammonia (32%) and then stirring at 20 °C for 18 h. The solution was concentrated under reduced pressure and using column chromatography was eluted with DCM in MeOH (9.5:05 v/v). After combining fractions and concentrating them, the desired product **14** was obtained as a colorless oil (0.57 g, 46%), homogeneous by TLC analysis (*R*_f_ = 0.6, DCM/MeOH/aq. ammonia (32%), 50:10:1 v/v/v). HRMS: found 503.3728 (*m*/*z*), C_25_H_51_N_4_O_6_ requires 503.3730 (*m*/*z*) [M + H]^+^; IR (film): 1692 (C=O) cm^–1^; ^1^H NMR, 500 MHz (CDCl_3_): 1.41–1.53 (m, 31H, 9× CH_3_, Boc, 6-CH_2_, 7-CH_2_, overlapping), 1.60–1.65 (m, 4H, 2-CH_2_, 11-CH_2_), 1.70–1.75 (s, 2H, NH_2_), 2.70 (t, *J* = 6.7 Hz, 2H, 12-CH_2_), 3.04–3.29 (m, 10H, 1-CH_2_, 3-CH_2_, 5-CH_2_, 8-CH_2_, 10-CH_2_, overlapping); ^13^C NMR, 125.77 MHz (CDCl_3_): 25.5, 25.8 (6-CH_2_, 7-CH_2_), 28.4, 29.0, 34.0 (9× CH_3_, Boc, 2-CH_2_, 11-CH_2_, overlapping), 37.3 (12-CH_2_), 42.0, 42.2, 43.9, 44.1 (1-CH_2_, 3-CH_2_, 5-CH_2_, 8-CH_2_, 10-CH_2_, overlapping), 79.2–81.4 (3× Cq, Boc), 156.1–157.9 (3× C=O, Boc).^10−12^

#### Succinic Acid (*N*^1^,*N*^4^,*N*^17^-Tri-*tert*-butoxycarbonyl)-1,20-diamino-4,17-diazaicosanyl Half Amide 15

A solution of the tri-Boc protected polyamine **12** (0.50 g, 0.81 mmol) in anhydrous pyridine (5 mL) under nitrogen was treated with succinic anhydride (0.08 g, 0.81 mmol, 1 equiv) at 20 °C. The solution was stirred for a further 18 h. The solution was then concentrated in vacuo, and the crude material was extracted with chloroform (3× 15 mL). The combined organic extracts were dried (Na_2_SO_4_), filtered, and concentrated in vacuo. The desired product **15** was obtained as a colorless oil (0.42 g, 72%), homogeneous by TLC analysis (*R*_f_ = 0.6, EtOAc/EtOH/aq. ammonia (32%); 7:2:1 v/v/v). HRMS: found 713.5182 (*m*/*z*), C_37_H_69_N_4_O_9_ requires 713.5143 (*m*/*z*) [M – H]^−^; IR (film): 2892–3736 (COOH) and 1686 (C=O) cm^–1^; ^1^H NMR, 500 MHz (CDCl_3_): 1.24–1.35 (m, 16H, 7-CH_2_, 8-CH_2_, 9-CH_2_, 10-CH_2_, 11-CH_2_, 12-CH_2_, 13-CH_2_, 14-CH_2_), 1.44–158 (m, 31H, 9× CH_3_, Boc, 6-CH_2_, 15-CH_2_, overlapping), 1.75–1.88 (m, 4H, 2-CH_2_, 19-CH_2_, overlapping), 2.54 (t, *J* = 7.0 Hz, 2H, CH_2_), 2.77 (t, *J* = 7.0 Hz, 2H, CH_2_), 3.04–3.36 (m, 12H, 1-CH_2_, 3-CH_2_, 5-CH_2_, 16-CH_2_, 18-CH_2_, 20-CH_2_, overlapping); ^13^C NMR, 125.77 MHz (CDCl_3_): 24.5 (19-CH_2_), 26.8, 27.0 (9-CH_2_, 10-CH_2_, 11-CH_2_, 12-CH_2_, overlapping), 28.5, 28.8 (9× CH_3_, Boc, 2-CH_2_, 6-CH_2_, 15-CH_2_, overlapping), 29.3, 29.9 (7-CH_2_, 8-CH_2_, 13-CH_2_, 14-CH_2_, overlapping), 30.7 (CH_2_), 32.7 (CH_2_), 37.1, 42.4, 44.0, 45.5, 47.0 (1-CH_2_, 3-CH_2_, 5-CH_2_, 16-CH_2_, 18-CH_2_, 20-CH_2_, overlapping), 79.8–81.1 (3× Cq, Boc), 155.7–157.1 (3× C=O, Boc), 172.4 (CONH), 174.2 (COOH).

#### Succinic Acid (*N*^1^,*N*^4^,*N*^8^-Tri-*tert*-butoxycarbonyl)-1,11-diamino-4,8-diazaundecanyl Half Amide 16

A solution of the tri-Boc protected norspermine (thermine, 3.3.3) **13** (0.35 g, 0.71 mmol) in anhydrous pyridine (5 mL) under nitrogen was treated with succinic anhydride (0.07 g, 0.71 mmol, 1 equiv) at 20 °C. The solution was stirred for a further 18 h. The solution was then concentrated in vacuo, and the crude material was extracted with chloroform (3× 15 mL). The combined organic extracts were dried (Na_2_SO_4_), filtered, and concentrated in vacuo. The desired product **16** was obtained as a colorless oil (0.31 g, 75%), homogeneous by TLC analysis (*R*_f_ = 0.3, EtOAc/EtOH/aq. ammonia (32%); 7:2:1 v/v/v). HRMS: found 587.3730 (*m*/*z*), C_28_H_51_N_4_O_9_ requires 587.3734 (*m*/*z*) [M – H]^−^; IR (film): 3074–3746 (COOH), and 1629 (C=O), cm^–1^; ^1^H NMR, 500 MHz (CD_3_OD): 1.42–1.48 (m, 27H, 9× CH_3_, Boc), 1.64–1.80 (m, 6H, 2-CH_2_, 6-CH_2_, 10-CH_2_, overlapping), 2.51 (t, *J* = 7.0 Hz, 2H, CH_2_), 2.68 (t, *J* = 7.0 Hz, 2H, CH_2_), 3.03–3.24 (m, 12H, 1-CH_2_, 3-CH_2_, 5-CH_2_, 7-CH_2_, 9-CH_2_, 11-CH_2_, overlapping); ^13^C NMR, 125.77 MHz (CD_3_OD): 27.5 (9× CH_3_, Boc, 2-CH_2_, 6-CH_2_, 10-CH_2_, overlapping), 29.6 (CH_2_), 30.5 (CH_2_), 35.4, 36.7, 43.2, 44.3 (1-CH_2_, 3-CH_2_, 5-CH_2_, 7-CH_2_, 9-CH_2_, 11-CH_2_, overlapping), 79.2–80.9 (3× Cq, Boc), 155.2–157.4 (3× C=O, Boc), 171.2 (CONH), 173.9 (COOH).

#### Succinic Acid (*N*^1^,*N*^4^,*N*^9^-Tri-*tert*-butoxycarbonyl)-1,12-diamino-4,9-diazadodecanyl Half Amide 17

A solution of the tri-Boc protected spermine **14** (0.61 g, 1.21 mmol) in anhydrous pyridine (5 mL) under nitrogen was treated with succinic anhydride (0.12 g, 1.21 mmol, 1 equiv) at 20 °C. The solution was stirred for a further 18 h. The solution was then concentrated in vacuo. The crude material was extracted with chloroform (3× 15 mL). The combined organic extracts were dried (Na_2_SO_4_), filtered, and concentrated in vacuo. The desired product **17** was obtained as a colorless oil (0.63 g, 86%). Homogeneous by TLC analysis (*R*_f_ = 0.4, EtOAc/EtOH/aq. ammonia (32%); 7:2:1 v/v/v). HRMS: found 601.3867 (*m*/*z*), C_29_H_53_N_4_O_9_ requires 601.3860 (*m*/*z*) [M – H]^−^; IR (film): 2948–3536 (COOH) and 1686 (C=O) cm^–1^; ^1^H NMR, 500 MHz (CD_3_OD): 1.41–1.49 (m, 27H, 9× CH_3_, Boc) 1.48–1.53 (m, 4H, 6-CH_2_, 7-CH_2_), 1.64–1.74 (m, 4H, 2-CH_2_, 11-CH_2_), 2.45 (t, *J* = 7.0 Hz, 2H, CH_2_), 2.58 (t, *J* = 7.0 Hz, 2H, CH_2_), 3.03–3.24 (m, 12H, 1-CH_2_, 3-CH_2_, 5-CH_2_, 8-CH_2_, 10-CH_2_, 12-CH_2_, overlapping); ^13^C NMR, 125.77 MHz (CD_3_OD): 25.5, 26.8 (6-CH_2_, 7-CH_2_), 27.5 (9× CH_3_, Boc), 26.9 (2-CH_2_, 10-CH_2_), 28.8 (CH_2_), 30.26 (CH_2_), 36.3, 36.7, 39.9, 44.1 (1-CH_2_, 3-CH_2_, 5-CH_2_, 8-CH_2_, 10-CH_2_, 12-CH_2_, overlapping), 79.5–81.0 (3× Cq, Boc), 156.1–157.5 (3× C=O, Boc), 173.4 (CONH), 174.8 (COOH).

#### Succinic Acid Di-(*N*^1^,*N*^4^,*N*^17^-tri-*tert*-butoxycarbonyl)-1,20-diamino-4,17-diazaicosanyl Amide 18

A solution of succinic acid half amide **15** (0.17 g, 0.24 mmol), HBTu (0.09 g, 0.24 mmol, 1 equiv), and TEA (0.07 g, 0.02 mmol, 1 equiv) in anhydrous DMF (10 mL) was treated with **12** (0.15 g, 0.24 mmol, 1 equiv) in anhydrous DMF (3 mL) under nitrogen at 20 °C. The solution was stirred for a further 18 h. The solution was then concentrated in vacuo. The crude material was extracted with chloroform (3 × 15 mL). The combined organic extracts were dried (Na_2_SO_4_), filtered, concentrated in vacuo, and purified over silica gel (DCM/MeOH; 9.9:0.1 to 9:1 v/v). After combining fractions and concentrating them, the desired product **18** was obtained as a pale yellow oil (0.24 g, 75%), homogeneous by TLC analysis (*R*_f_ = 0.5, DCM: MeOH, 9:1 v/v). HRMS: found 1333.9990 (*m*/*z*), C_70_H_134_NaN_8_O_14_ requires 1334.0020 (*m*/*z*) [M + Na]^+^; IR (film): 1686 (C=O) cm^–1^; ^1^H NMR, 500 MHz (CDCl_3_): 1.26 (s, 40H, 6-CH_2_, 7-CH_2_, 8-CH_2_, 9-CH_2_, 10-CH_2_, 11-CH_2_, 12-CH_2_, 13-CH_2_, 14-CH_2_, 15-CH_2_), 1.38–1.51 (m, 54H, 18× CH_3_, Boc), 1.59–1.72 (m, 8H, 2-CH_2_, 19-CH_2_), 2.53 (s, 4H, 23-CH_2_), 3.00–3.32 (m, 24H, 1-CH_2_, 3-CH_2_, 5-CH_2_, 16-CH_2_, 18-CH_2_, 20-CH_2_); ^13^C NMR, 125.77 MHz (CDCl_3_): 26.6, 27.8, 28.7, 29.8 (18× CH_3_, Boc, 2-CH_2_, 6-CH_2_, 7-CH_2_, 8-CH_2_, 9-CH_2_, 10-CH_2_, 11-CH_2_, 12-CH_2_, 13-CH_2_, 14-CH_2_, 15-CH_2_, 19-CH_2_), 31.9 (23-CH_2_), 36.0, 37.5, 43.6, 47.2 (1-CH_2_, 3-CH_2_, 5-CH_2_, 16-CH_2_, 18-CH_2_, 20-CH_2_), 79.7, 80.7 (6× Cq, Boc), 156.4–156.9 (6× C=O, Boc), 172.6 (2× NHCO).

#### Succinic Acid (*N*^1^,*N*^4^,*N*^8^-Tri-*tert*-butoxycarbonyl)-1,11-diamino-4,8-diazaundecanyl (*N*^1^,*N*^4^,*N*^17^-Tri-*tert*-butoxycarbonyl)-1,20-diamino-4,17-diazaicosanyl Amide 19

A solution of succinic acid half amide **16** (0.29 g, 0.41 mmol), HBTu (0.15 g, 0.41 mmol, 1 equiv), and TEA (0.04 g, 0.41 mmol, 1 equiv) in anhydrous DMF (10 mL) was treated with **12** (0.20 g, 0.41, 1 equiv) in anhydrous DMF (3 mL) under nitrogen at 20 °C. The solution was stirred for a further 18 h. The solution was then concentrated in vacuo. The crude material was extracted with chloroform (3 × 15 mL). The combined organic extracts were dried (Na_2_SO_4_), filtered, concentrated in vacuo, and purified over silica gel (DCM/MeOH; 9.9:0.1 to 9:1 v/v). After combining fractions and concentrating them, the desired product **19** was obtained as a pale yellow oil (0.23 g, 48%), homogeneous by TLC analysis (*R*_f_ = 0.5, DCM/MeOH, 9:1 v/v). HRMS: found 1185.8698 (*m*/*z*), C_61_H_117_N_8_O_14_ requires 1185.8611 (*m*/*z*) [M + H]^+^; IR (film): 1687 (C=O) cm^–1^; ^1^H NMR, 500 MHz (CDCl_3_): 1.24 (s, 20H, 6-CH_2_, 7-CH_2_, 8-CH_2_, 9-CH_2_, 10-CH_2_, 11-CH_2_, 12-CH_2_, 13-CH_2_, 14-CH_2_, 15-CH_2_), 1.36–1.50 (m, 54H, 18× CH_3_, Boc), 1.58–1.77 (m, 10H, 2-CH_2_, 19-CH_2_, 28-CH_2_, 32-CH_2_, 36-CH_2_), 2.52 (s, 4H, 23-CH_2_, 24-CH_2_), 3.03–3.30 (m, 24H, 1-CH_2_, 3-CH_2_, 5-CH_2_, 16-CH_2_, 18-CH_2_, 20-CH_2_, 27-CH_2_, 29-CH_2_, 31-CH_2_, 33-CH_2_, 35-CH_2_, 37-CH_2_); ^13^C NMR, 125.77 MHz (CDCl_3_): 26.9, 27.8, 28.6, 29.6 (18× CH_3_, Boc, 2-CH_2_, 6-CH_2_, 7-CH_2_, 8-CH_2_, 9-CH_2_, 10-CH_2_, 11-CH_2_, 12-CH_2_, 13-CH_2_, 14-CH_2_, 15-CH_2_, 19-CH_2_, 28-CH_2_, 32-CH_2_, 36-CH_2_), 31.8 (23-CH_2_, 24-CH_2_), 36.2, 37.7, 38.7, 43.7, 45.0, 47.1 (1-CH_2_, 3-CH_2_, 5-CH_2_, 16-CH_2_, 18-CH_2_, 20-CH_2_, 27-CH_2_, 29-CH_2_, 31-CH_2_, 33-CH_2_, 35-CH_2_, 37-CH_2_), 79.7–80.8 (6× Cq, Boc), 156.4–157.6 (6× C=O, Boc), 172.6 (2× NHCO).

#### Succinic Acid (*N*^1^,*N*^4^,*N*^9^-Tri-*tert*-butoxycarbonyl)-1,12-diamino-4,9-diazadodecanyl (*N*^1^,*N*^4^,*N*^17^-Tri-*tert*-butoxycarbonyl)-1,20-diamino-4,17-diazaicosanyl Amide 20

A solution of succinic acid half amide **17** (0.42 g, 0.59 mmol), HBTu (0.22 g, 0.59 mmol, 1 equiv), and TEA (0.05 g, 0.59 mmol, 1 equiv) in anhydrous DMF (10 mL) was treated with **12** (0.35 g, 0.59 mmol, 1 equiv) in anhydrous DMF (3 mL) under nitrogen at 20 °C and then stirred for a further 18 h. The solution was then concentrated in vacuo, and the crude material was extracted with chloroform (3 × 15 mL). The combined organic extracts were dried (Na_2_SO_4_), filtered, concentrated in vacuo, and purified over silica gel (DCM/MeOH; 9.5:0.5 to 9:1 v/v). After combining fractions and concentrating them, the desired product **20** was obtained as a pale yellow oil (0.28 g, 37%), homogeneous by TLC analysis (*R*_f_ = 0.6 (DCM/MeOH, 9:1 v/v). HRMS: found 1221.8624 (*m*/*z*), C_62_H_118_NaN_8_O_14_ requires 1221.8768 (*m*/*z*) [M + Na]^+^; IR (film): 1663 (C=O) cm^–1^; ^1^H NMR, 500 MHz (CDCl_3_): 1.26 (m, 20H, 6-CH_2_, 7-CH_2_, 8-CH_2_, 9-CH_2_, 10-CH_2_, 11-CH_2_, 12-CH_2_, 13-CH_2_, 14-CH_2_, 15-CH_2_), 1.39–1.53 (m, 58H, 18× CH_3_, Boc, 32-CH_2_, 33-CH_2_), 1.61–1.73 (m, 8H, 2-CH_2_, 19-CH_2_, 28-CH_2_, 37-CH_2_), 2.52 (s, 4H, 23-CH_2_, 24-CH_2_), 3.03–3.37 (m, 24H, 1-CH_2_, 3-CH_2_, 5-CH_2_, 16-CH_2_, 18-CH_2_, 20-CH_2_, 27-CH_2_, 29-CH_2_, 31-CH_2_, 34-CH_2_, 36-CH_2_, 38-CH_2_); ^13^C NMR, 125.77 MHz (CDCl_3_): 25.8, 27.4, 28.3, 28.6 (18× CH_3_, Boc, 2-CH_2_, 6-CH_2_, 7-CH_2_, 8-CH_2_, 9-CH_2_, 10-CH_2_, 11-CH_2_, 12-CH_2_, 13-CH_2_, 14-CH_2_, 15-CH_2_, 19-CH_2_, 28-CH_2_, 32-CH_2_, 33-CH_2_, 37-CH_2_), 30.8 (23-CH_2_, 24-CH_2_), 34.9, 36.1, 37.6, 43.5, 46.0 (1-CH_2_, 3-CH_2_, 5-CH_2_, 16-CH_2_, 18-CH_2_, 20-CH_2_, 27-CH_2_, 29-CH_2_, 31-CH_2_, 34-CH_2_, 36-CH_2_, 38-CH_2_), 79.3–81.6 (6× Cq, Boc), 156.0–157.7 (6× C=O, Boc), 173.6 (2× NHCO).

#### Succinic Acid Di-1,20-diamino-4,17-diazaicosanyl Amide 21

HexaBoc succinic acid amide **18** (0.12 g, 0.09 mmol) was deprotected according to general procedure A to yield the desired product **21** as a pale yellow oil (0.12 g, 99%). HRMS: found 733.6757 (*m*/*z*), C_40_H_86_NaN_8_O_2_ requires 733.6874 (*m*/*z*) [M + Na]^+^; IR (film): 1607 (C=O) and 2963–3006 (NH, NH_2_^+^, NH_3_^+^) cm^–1^; ^1^H NMR, 500 MHz (D_2_O): 1.20–1.37 (m, 32H, 7-CH_2_, 8-CH_2_, 9-CH_2_, 10-CH_2_, 11-CH_2_, 12-CH_2_, 13-CH_2_, 14-CH_2_), 1.58–1.68, (m, 8H, 6-CH_2_, 15-CH_2_), 1.80–1.89 (m, 4H, 19-CH_2_), 2.00–2.10 (m, 4H, 2-CH_2_), 2.51 (s, 4H, 23-CH_2_), 2.94–3.13 (m, 20H, 1-CH_2_, 3-CH_2_, 5-CH_2_, 16-CH_2_, 18-CH_2_), 3.24 (t, *J* = 6.7 Hz, 4H, 20-CH_2_); ^13^C NMR, 125.77 MHz (D_2_O): 23.6 (19-CH_2_), 25.45, 25.55, 25.63, 28.1, 28.4, 28.6 (2-CH_2_, 6-CH_2_, 7-CH_2_, 8-CH_2_, 9-CH_2_, 10-CH_2_, 11-CH_2_, 12-CH_2_, 13-CH_2_, 14-CH_2_, 15-CH_2_), 30.6 (23-CH_2_), 35.9 (20-CH_2_), 36.5, 44.3, 44.7, 47.7, 47.8 (1-CH_2_, 3-CH_2_, 5-CH_2_, 16-CH_2_, 18-CH_2_), 116.4 (q, ^1^*J* = 292.4 Hz, CF_3_), 160.8 (q, ^2^*J* = 36.1 Hz, CO–CF_3_), 175.1 (2× NHCO).

#### Succinic Acid 1,11-Diamino-4,8-diazaundecanyl 1,20-Diamino-4,17-diazaicosanyl Amide 22

HexaBoc succinic acid amide **19** (0.11 g, 0.09 mmol) was deprotected according to general procedure A to yield the desired product **22** as a pale yellow oil (0.11 g, 99%). HRMS: found 585.5538 (*m*/*z*), C_31_H_69_N_8_O_2_ requires 585.5465 (*m*/*z*) [M + H]^+^; IR (film): 1688 (C=O); ^1^H NMR, 500 MHz (D_2_O): 1.20–1.38 (m, 16H, 7-CH_2_, 8-CH_2_, 9-CH_2_, 10-CH_2_, 11-CH_2_, 12-CH_2_, 13-CH_2_, 14-CH_2_), 1.58–1.69 (m, 4H, 6-CH_2_, 15-CH_2_), 1.80–1.90 (m, 4H, 19-CH_2_, 28-CH_2_), 1.99–2.13 (m, 6H, 2-CH_2_, 32-CH_2_, 36-CH_2_), 2.51 (s, 4H, 23-CH_2_, 24-CH_2_), 2.92–3.17 (m, 20H, 1-CH_2_, 3-CH_2_, 5-CH_2_, 16-CH_2_, 18-CH_2_, 29-CH_2_, 31-CH_2_, 33-CH_2_, 35-CH_2_, 37-CH_2_), 3.23 (t, *J* = 7.6 Hz, 4H, 20-CH_2_, 27-CH_2_); ^13^C NMR, 125.77 MHz (D_2_O): 22.6 (2-CH_2_, 32-CH_2_, 36-CH_2_), 25.6, 28.1, 28.4, 28.6 (6-CH_2_, 7-CH_2_, 8-CH_2_, 9-CH_2_, 10-CH_2_, 11-CH_2_, 12-CH_2_, 13-CH_2_, 14-CH_2_, 15-CH_2_, 19-CH_2_, 28-CH_2_, overlapping), 30.6 (23-CH_2_, 24-CH_2_), 36.0 (20-CH_2_, 27-CH_2_), 36.4, 44.1, 44.9, 45.3, 47.9 (1-CH_2_, 3-CH_2_, 5-CH_2_, 16-CH_2_, 18-CH_2_, 29-CH_2_, 31-CH_2_, 33-CH_2_, 35-CH_2_, 37-CH_2_), 116.5 (q, ^1^*J* = 290.9 Hz, CF_3_), 162.6 (q, ^2^*J* = 37.1 Hz, CO–CF_3_), 175.1 (2× NHCO).

#### Succinic Acid 1,12-Diamino-4,9-diazadodecanyl 1,20-Diamino-4,17-diazaicosanyl Amide 23

HexaBoc succinic acid amide **20** (0.28 g, 0.22 mmol) was deprotected according to general procedure A to yield the desired product **23** as a pale yellow oil (0.28 g, 99%). HRMS: found 599.5690 (*m*/*z*), C_32_H_71_N_8_O_2_ requires 599.5630 (*m*/*z*) [M + H]^+^; IR (Film): 1676 (C=O); ^1^H NMR, 500 MHz (D_2_O): 1.22–1.37 (m, 16H, 7-CH_2_, 8-CH_2_, 9-CH_2_, 10-CH_2_, 11-CH_2_, 12-CH_2_, 13-CH_2_, 14-CH_2_), 1.60–1.69 (m, 4H, 6-CH_2_, 15-CH_2_), 1.72–1.78 (m, 4H, 32-CH_2_, 33-CH_2_), 1.82–1.91 (m, 4H, 19-CH_2_, 28-CH_2_), 2.00–2.13 (m, 4H, 2-CH_2_, 37-CH_2_), 2.52 (s, 4H, 23-CH_2_, 24-CH_2_), 2.95–3.16 (m, 20H, 1-CH_2_, 3-CH_2_, 5-CH_2_, 16-CH_2_, 18-CH_2_, 29-CH_2_, 31-CH_2_, 34-CH_2_, 36-CH_2_, 38-CH_2_), 3.26 (t, *J* = 7.2 Hz, 4H, 20-CH_2_, 27-CH_2_); ^13^C NMR, 125.77 MHz (D_2_O): 22.5 (32-CH_2_, 33-CH_2_), 23.8 (2-CH_2_, 37-CH_2_), 25.5, 27.9, 28.1, 28.4, 28.6 (6-CH_2_, 7-CH_2_, 8-CH_2_, 9-CH_2_, 10-CH_2_, 11-CH_2_, 12-CH_2_, 13-CH_2_, 14-CH_2_, 15-CH_2_, 19-CH_2_, 28-CH_2_, overlapping), 30.7 (23-CH_2_, 24-CH_2_), 36.2 (20-CH_2_, 27-CH_2_), 36.5, 44.4, 44.8, 45.0, 46.8, 46.9, 47.7, 47.8 (1-CH_2_, 3-CH_2_, 5-CH_2_, 16-CH_2_, 18-CH_2_, 29-CH_2_, 31-CH_2_, 34-CH_2_, 36-CH_2_, 38-CH_2_), 116.0 (q, ^1^*J* = 292.6 Hz, CF_3_), 162.7 (q, ^2^*J* = 36.6 Hz, CO–CF_3_), 175.2 (2× NHCO).

## Supporting Information Available

Practical synthesis of long linear polyamines; IR spectrum of dinitrile **2**; ^13^C NMR spectra of compounds **2** and **3**; ^1^H-^13^C HMBC NMR spectrum of succinic acid half amide **17**; ^1^H NMR spectra of the succinic acid amide region in half acid amide **17** and diamide **20**; stacked ^1^H NMR spectra of the three products **21**, **22**, and **23**; ^13^C, ^1^H-^13^C HSQC, and ^1^H-^13^C HMBC NMR spectra of **21**, **22**, and **23**, respectively (PDF)
