# Supplementary material for: Practical Synthesis of Antimicrobial Long Linear Polyamine Succinamides
Source: ACS Bio Med Chem Au. 2022 Oct 11;2(6):607–16. doi: 10.1021/acsbiomedchemau.2c00033 (PMC10125363; doi:10.1021/acsbiomedchemau.2c00033)
Supplement: Supplementary file 1 — bg2c00033_si_001.pdf [file bg2c00033_si_001.pdf]

Supplementary information

Practical synthesis of antimicrobial long linear polyamine succinamides

Abdulaziz H. Alkhzem,[1] Shuxian Li,[2] Toska Wonfor,[2] Timothy J. Woodman,[1] Maisem Laabei,[2] and Ian S. Blagbrough *[1]

[1] *Department of Pharmacy and Pharmacology, University of Bath, Bath BA2 7AY, UK*

[2] *Department of Biology and Biochemistry, University of Bath, Bath BA2 7AY, UK*

* Corresponding author: Ian S. Blagbrough email: prsisb@bath.ac.uk

Practical synthesis of long linear polyamines

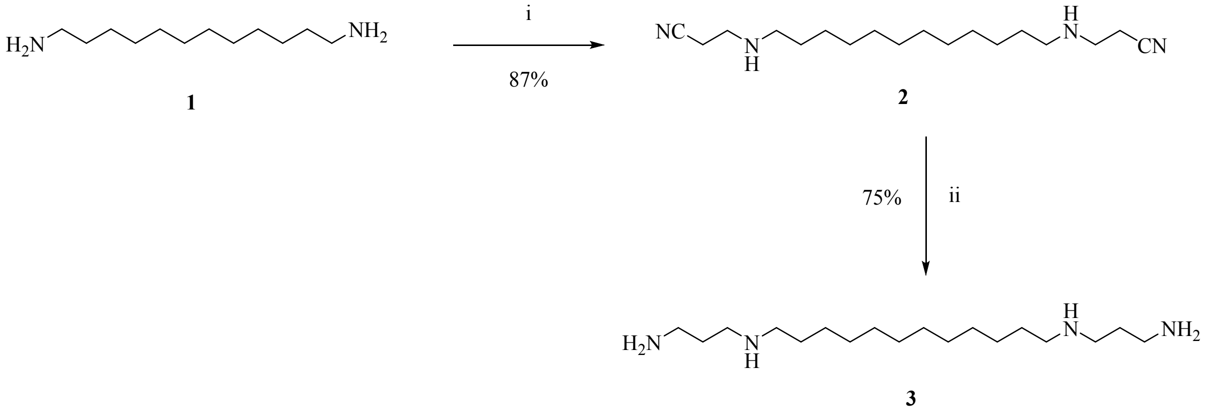

Fig. S1 *Reagents and conditions*: (i) Acrylonitrile, EtOH, 20 °C, 18 h; (ii) Raney Nickel, H_2, NaOH, EtOH, 20 °C, 18 h.

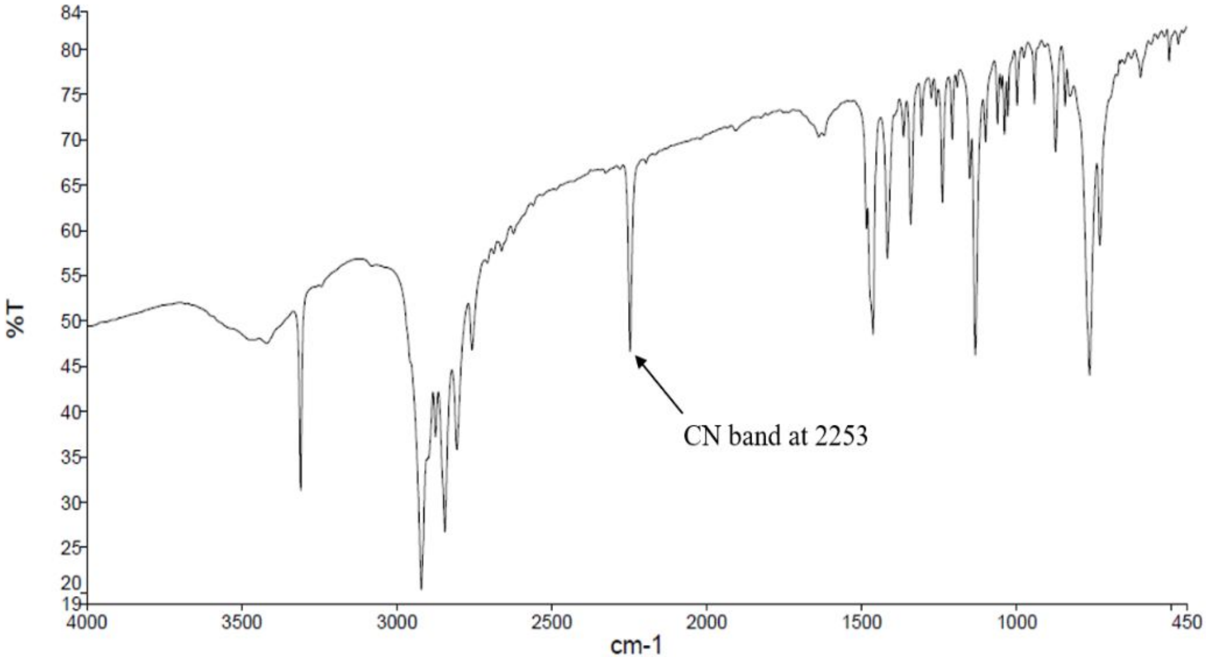

Fig. S2 Infrared (IR) spectrum of compound **2** showed the nitrile band at 2253 cm^{-1}.

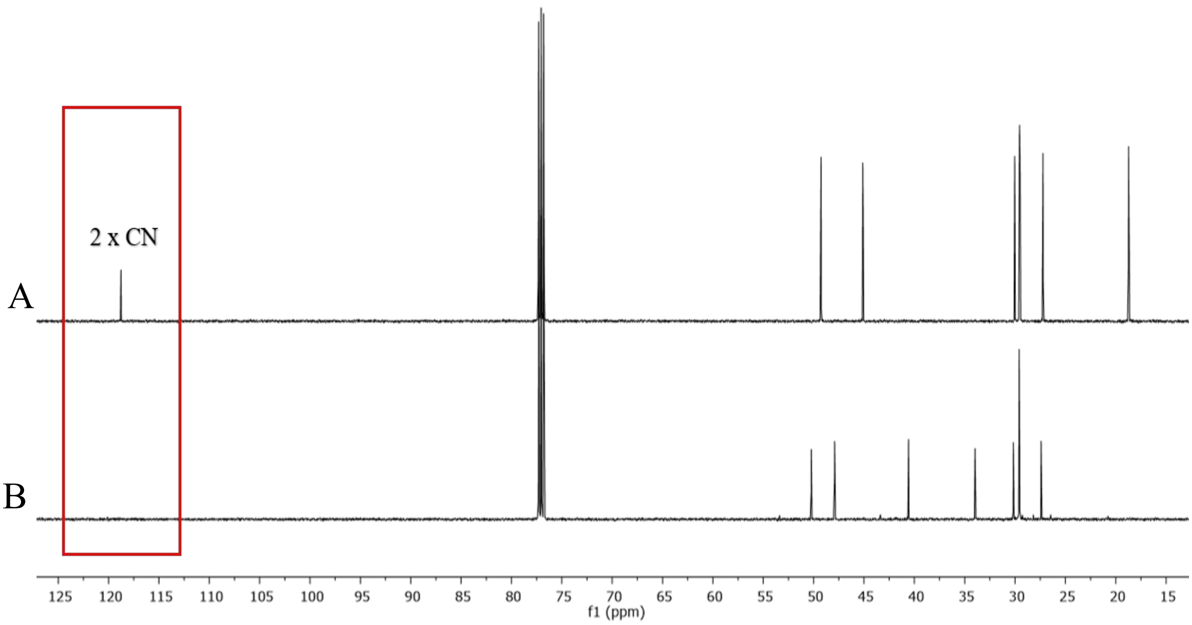

Fig. S3 ^{13}C NMR spectra of compounds **A 2** and **B 3** showed the loss of the nitrile peak upon catalytic hydrogenation measured relative to TMS in 99.8% $CDCl_3$ at 25 °C.

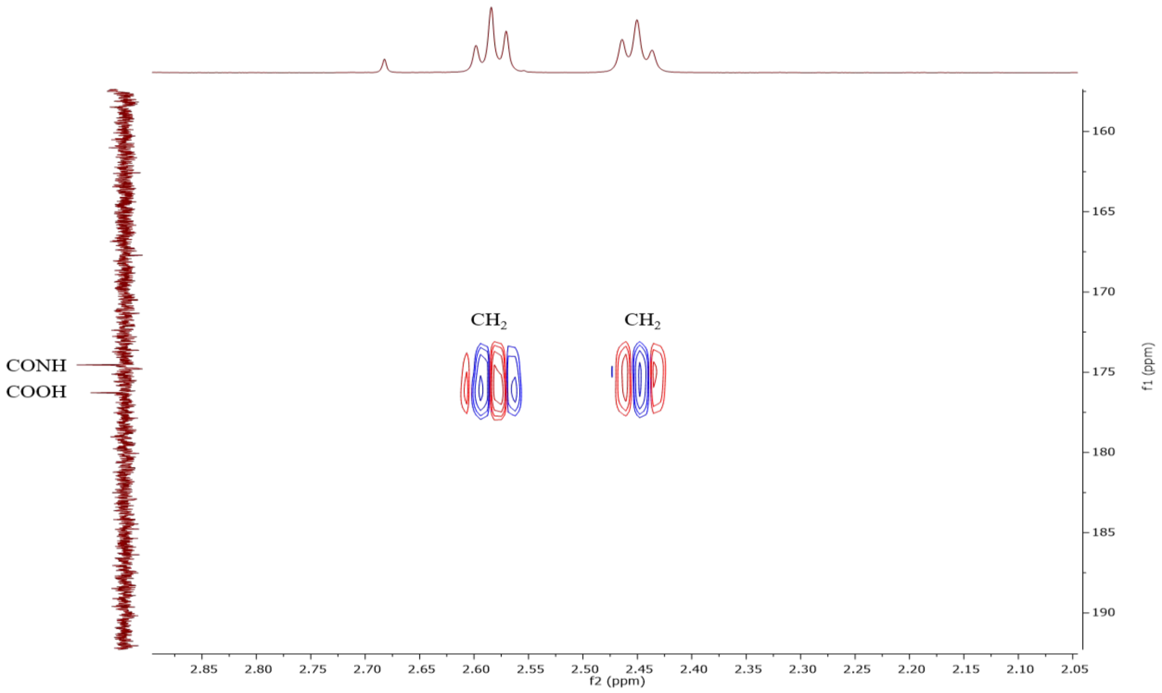

Fig. S4 ^{1}H-^{13}C HMBC NMR spectrum of compound **17** measured relative to TMS in 99.8% CD_3OD at 25 °C.

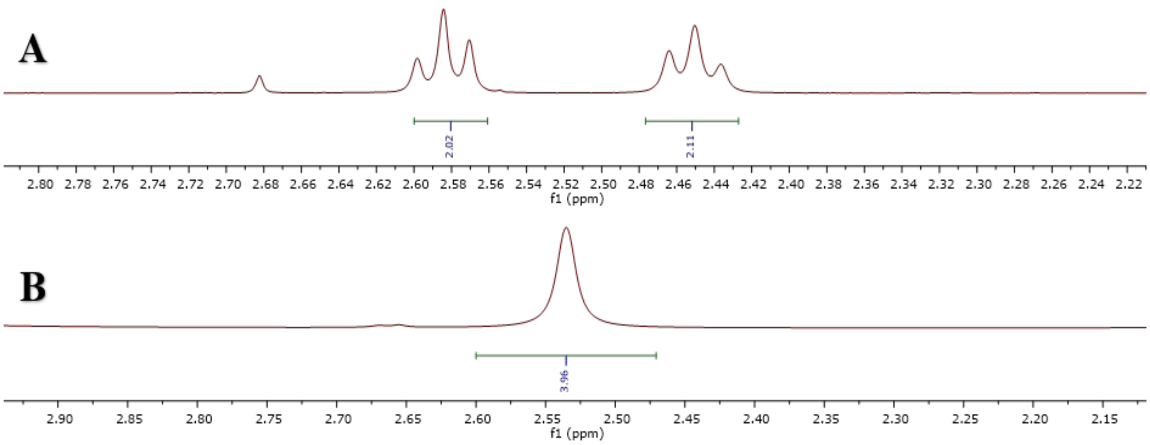

Fig. S5 The ^{1}H NMR spectra of **A 17** in 99.8% CD_3OD and **B 20** in 99.8% $CDCl_3$ measured relative to TMS at 25 °C.

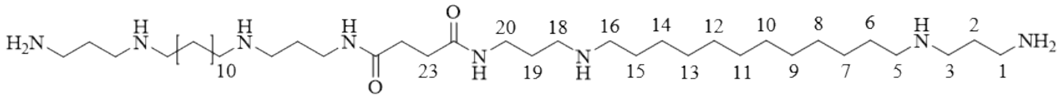

21

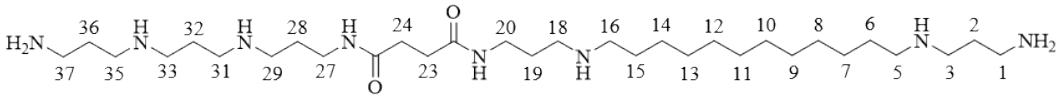

22

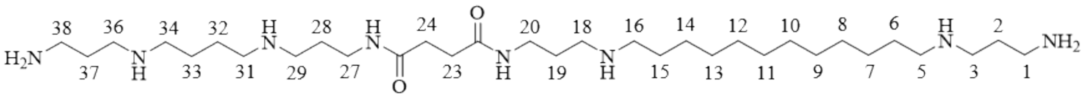

23

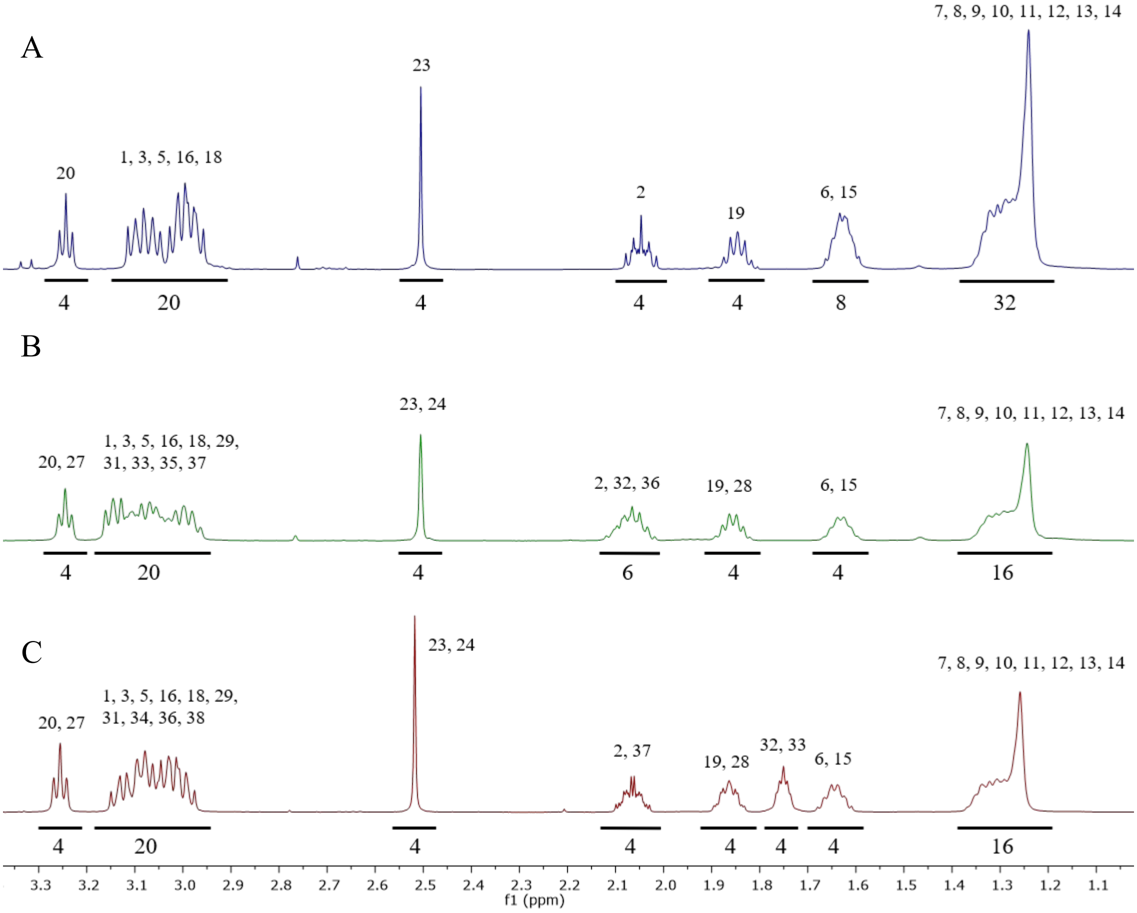

Fig. S6 Stacked ^{1}H NMR spectra of compounds **A 21**, **B 22**, and **C 23** as TFA salts measured relative to the residual solvent peak (HOD) at 4.79 ppm in 99.7% D_2O at 25 °C.

NMR spectroscopic assignments for homo- (21) and hetero- (22, and 23) dimeric linear polyamines incorporating a *N*^{1},*N*^{12}-di(3-aminopropyl)-1,12-diaminododecane moiety

The ^{1}H NMR spectroscopic resonances of methylene groups of poly-TFA salts of compounds **21**, **22**, and **23** can be observed in five distinct regions, around 3.2 ppm (t, J = 7.2 Hz) resonate methylene backbones adjacent to amide groups (20-CH_2 for compound **21**, 20-CH_2, 27-CH_2 for compounds **22**, and **23**), around 2.9-3.1 ppm methylene groups adjacent to primary and secondary amino groups (10 x NCH_2), around 2.5 ppm methylene groups which represent two methylene groups of the linker (RNHCO**CH_2CH_2**CONHR), around 2.0 and 1.8 ppm methylene groups separated from amino groups (NH_2 or/and NH) by one CH_2 group on each side (2-CH_2, 19-CH_2 for compound **21**, 2-CH_2, 19-CH_2, 28-CH_2, 32-CH_2, and 36-CH_2 for compound **22**, 2-CH_2, 19-CH_2, 28-CH_2, and 37-CH_2 for compound **23**), and around 1.6 and 1.3 ppm methylene groups separated from the secondary amines by one methylene group on one side and more than one methylene groups on the other side 6-CH_2, 15-CH_2 for compounds **21**, **22**, and **23** or more than on each side 7-CH_2, 8-CH_2, 9-CH_2, 10-CH_2, 11-CH_2, 12-CH_2, 13-CH_2, 14-CH_2 for compounds **21**, **22**, and **23**, see Suppl Fig. S6.

Methylene groups α to an amide (20-CH_2 for compound **21**, 20-CH_2, 27-CH_2 for compound **22**, and **23**) are more de-shielded, and therefore have the highest chemical resonance. The protonation of primary and secondary amine functional groups causes a de-shielding of the methylene functional group α to the nitrogen causing a downfield shift in their peaks. Therefore, CH_2 groups located next to an amine are more de-shielded than those located further away, 1-CH_2, 3-CH_2, 5-CH_2, 16-CH_2, 18-CH_2 for compound **21**, 1-CH_2, 3-CH_2, 5-CH_2, 16-CH_2, 18-CH_2, 29-CH_2, 31-CH_2, 33-CH_2, 35-CH_2, 37-CH_2 for compound **22**, and 1-CH_2, 3-CH_2, 5-CH_2, 16-CH_2, 18-CH_2, 29-CH_2, 31-CH_2, 34-CH_2, 36-CH_2, 38-CH_2 for compound **23**, see Suppl Fig. S6.

Methylene groups β to amide and secondary amines (19-CH_2 for compound **21**, 19-CH_2, 28-CH_2 for compounds **22** and **23**) are less de-shielded, and therefore have smaller chemical shifts than methylene groups β to primary and secondary or secondary and secondary amines (2-CH_2 for compound **21**, 2-CH_2, 32-CH_2, 36-CH_2 for compound **22**, and 2-CH_2, 37-CH_2 **23**). However,

protons 32-CH_2 and 33-CH_2 for compound **23** are β and γ to amines, for this reason they shifted slightly upfield in the ^{1}H NMR spectrum compared to methylene groups β to amide and secondary amines, see Suppl Fig. S6.

The most upfield chemical shifts were observed for methylene groups 6-CH_2, 15-CH_2, and 7-CH_2, 8-CH_2, 9-CH_2, 10-CH_2, 11-CH_2, 12-CH_2, 13-CH_2, 14-CH_2 for compounds **21**, **22**, and **23** as they are located further away from amines, however, due to the effect by the secondary amine from only one side, methylene groups 6-CH_2 and 15-CH_2, β to secondary amine, have larger chemical shifts than methylene groups 7-CH_2 to 14-CH_2, see Suppl Fig. S6.

In ^{13}C NMR spectra the magnetic resonance of methylene groups of poly-TFA salts of compounds **21**, **22**, and **23** can be found in four distinct regions, around 175 ppm representing the amide groups (2 x NH$\underline{C}$O for compounds **21**, **22**, and **23**), around 35-50 ppm methylene groups adjacent to primary amines, secondary amines and amid groups (12 x N$\underline{C}H_2$), around 30 ppm methylene groups which represent two methylene groups of the linker (RNHCO$\mathbf{\underline{C}H_2\underline{C}H_2}$CONHR), around 20-30 ppm methylene groups separated from amino groups (NH_2 or/and NH) by one CH_2 group on each side (2-$\underline{C}H_2$, 19-$\underline{C}H_2$ for compound **21**, 2-$\underline{C}H_2$, 19-$\underline{C}H_2$, 28-$\underline{C}H_2$, 32-$\underline{C}H_2$, and 36-$\underline{C}H_2$ for compound **22**, 2-$\underline{C}H_2$, 19-$\underline{C}H_2$, 28-$\underline{C}H_2$, and 37-$\underline{C}H_2$ for compound **23**), or methylene groups separated from the secondary amines by one CH_2 functional group on one side and more CH_2 groups on the other side (6-$\underline{C}H_2$, 15-$\underline{C}H_2$ for compounds **21**, **22**, and **23**), or more than one on each side (7-$\underline{C}H_2$, 8-$\underline{C}H_2$, 9-$\underline{C}H_2$, 10-$\underline{C}H_2$, 11-$\underline{C}H_2$, 12-$\underline{C}H_2$, 13-$\underline{C}H_2$, 14-$\underline{C}H_2$ for compounds **21**, **22**, and **23**).

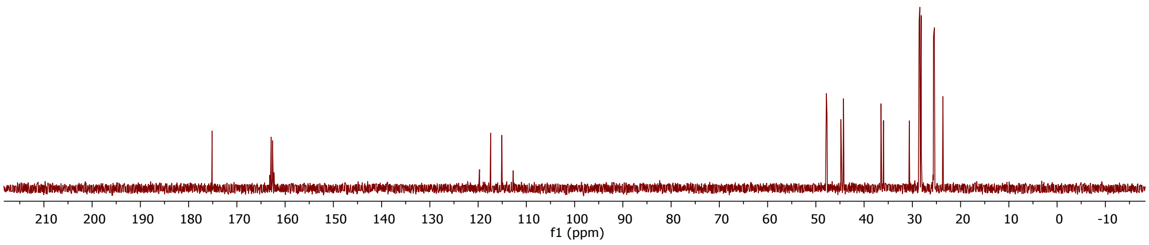

Fig. S7 ^{13}C NMR spectrum of compound **21** measured in 99.7% D_2O at 25 °C.

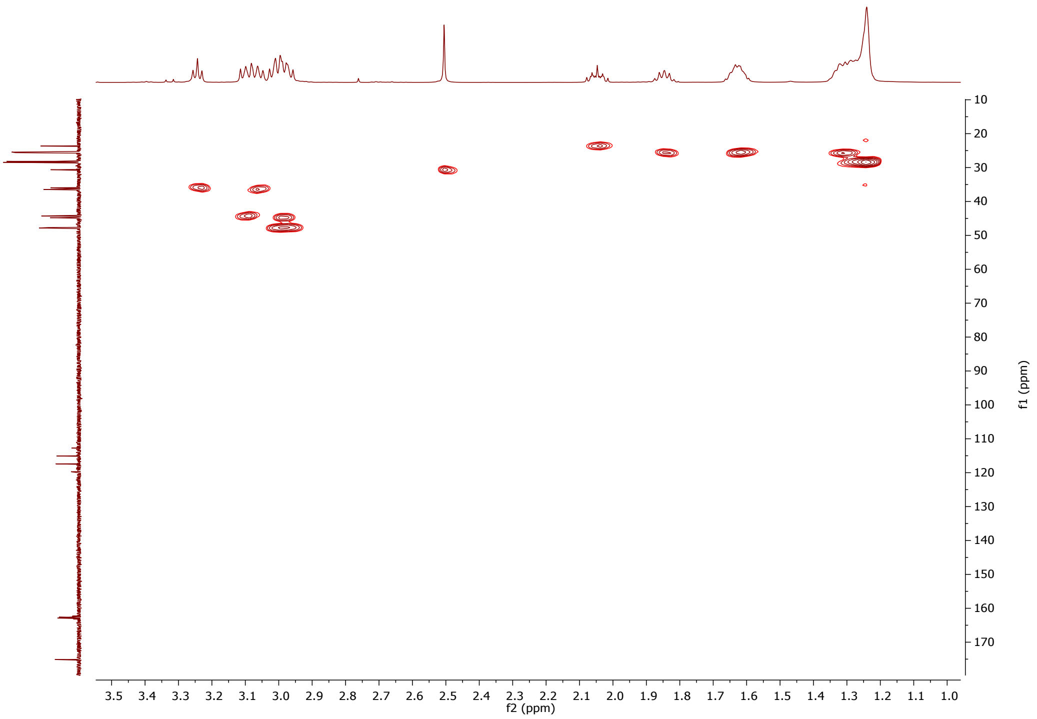

Fig. S8 ^{1}H-^{13}C HSQC NMR spectrum of compound **21** measured relative to the residual solvent peak (HOD) at 4.79 ppm in 99.7% D_2O at 25 °C.

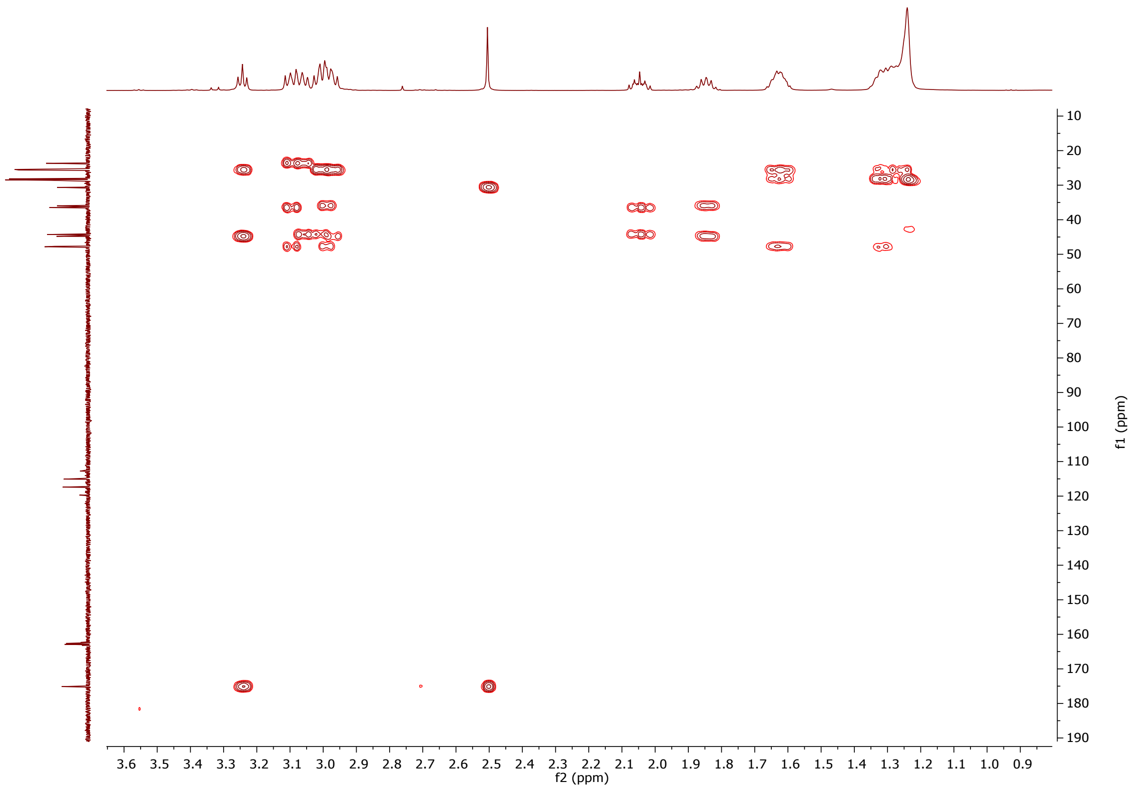

Fig. S9 ^{1}H-^{13}C HMBC NMR spectrum of compound **21** measured relative to the residual solvent peak (HOD) at 4.79 ppm in 99.7% D_2O at 25 °C.

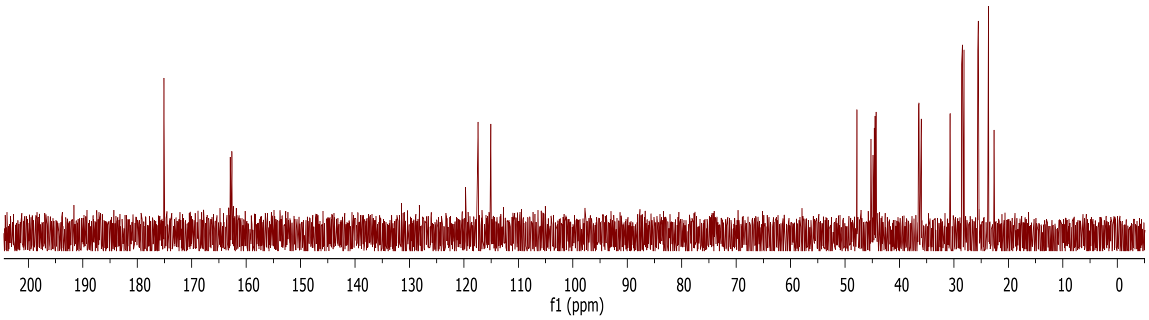

Fig. S10 ^{13}C NMR spectrum of compound **22** measured in 99.7% D_2O at 25 °C.

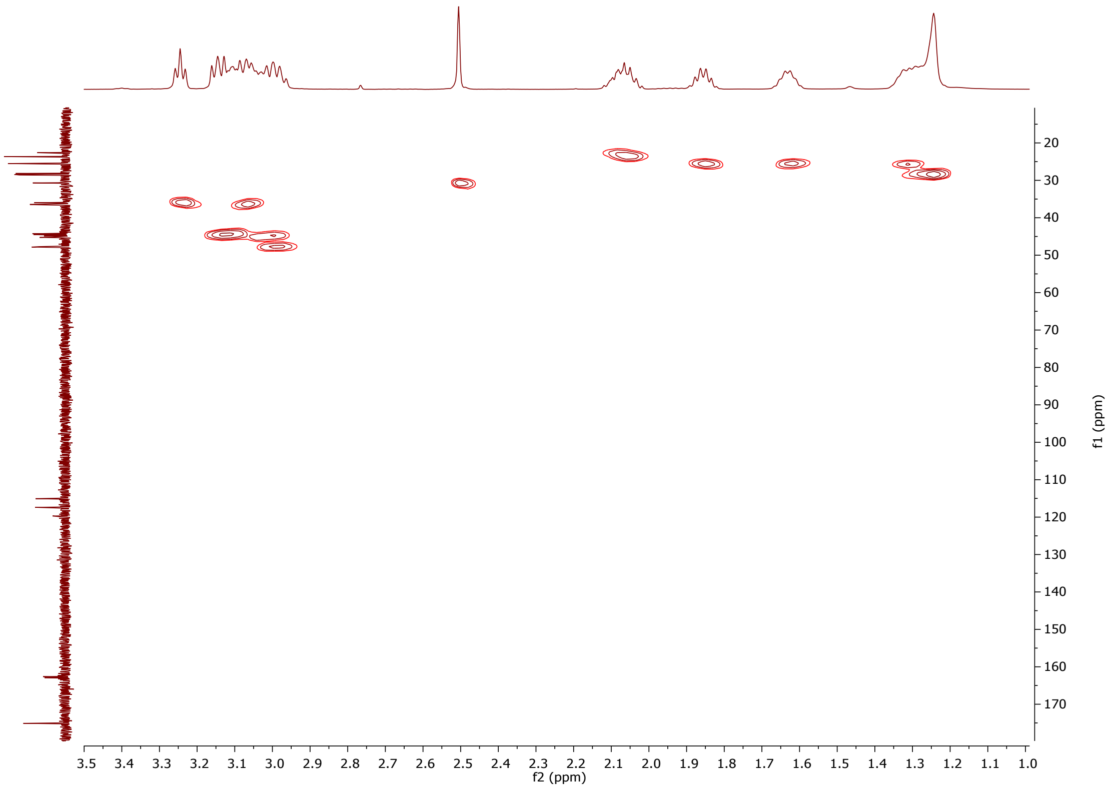

Fig. S11 ^{1}H-^{13}C HSQC NMR spectrum of compound **22** measured relative to the residual solvent peak (HOD) at 4.79 ppm in 99.7% D_2O at 25 °C.

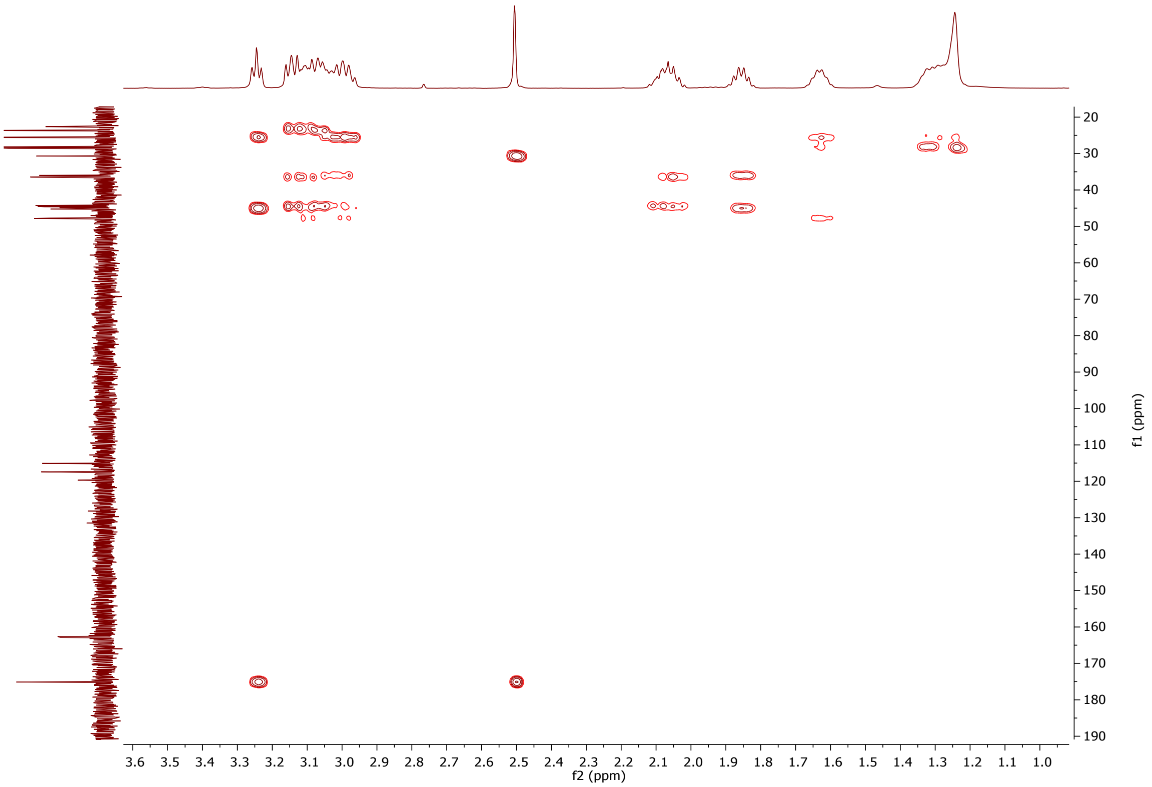

Fig. S12 ¹H-¹³C HMBC NMR spectrum of compound **22** measured relative to the residual solvent peak (HOD) at 4.79 ppm in 99.7% D_2O at 25 °C.

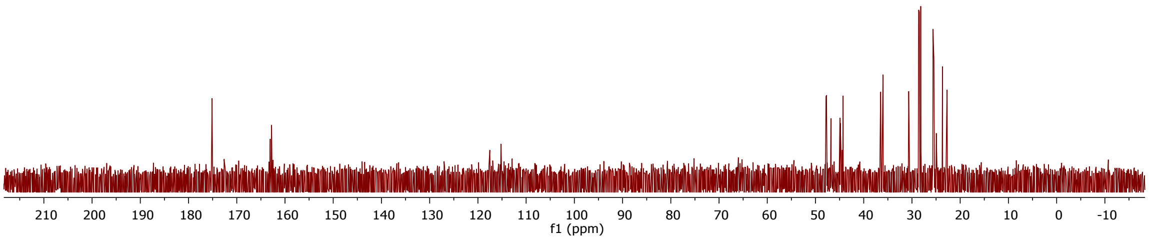

Fig. S13 ¹³C NMR spectrum of compound **23** measured in 99.7% D_2O at 25 °C.

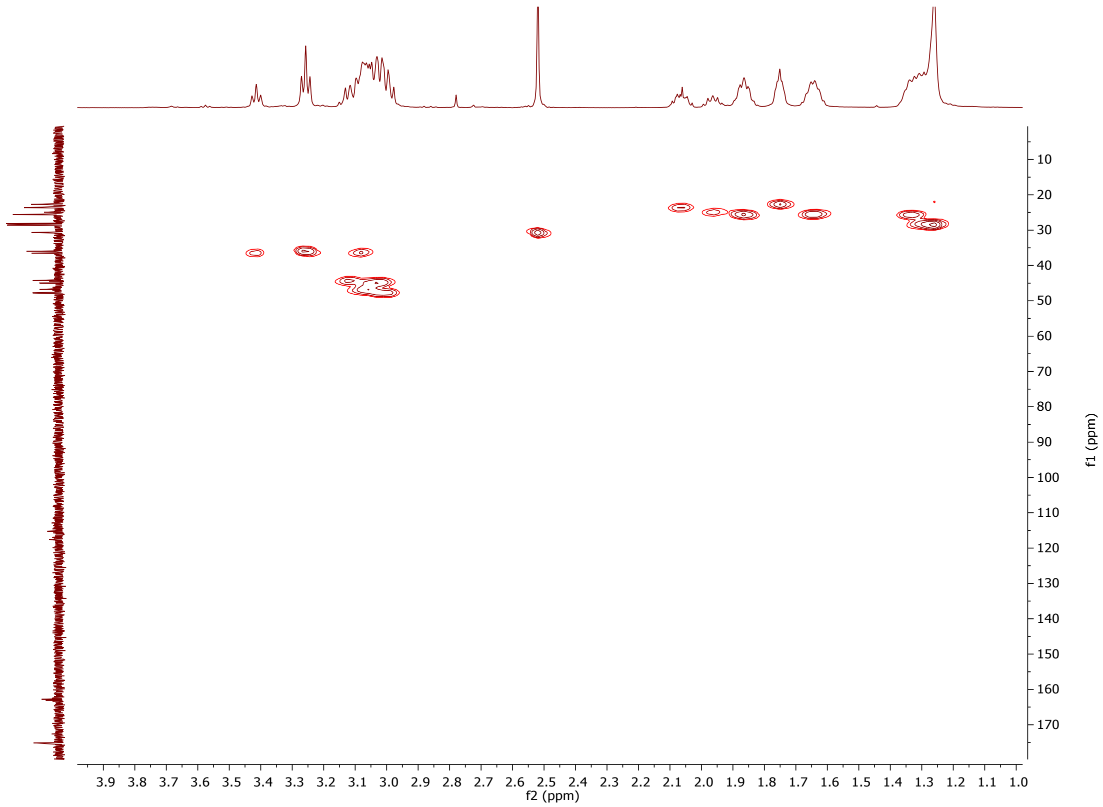

Fig. S14 ^{1}H-^{13}C HSQC NMR spectrum of compound **23** measured relative to the residual solvent peak (HOD) at 4.79 ppm in 99.7% D_2O at 25 °C.

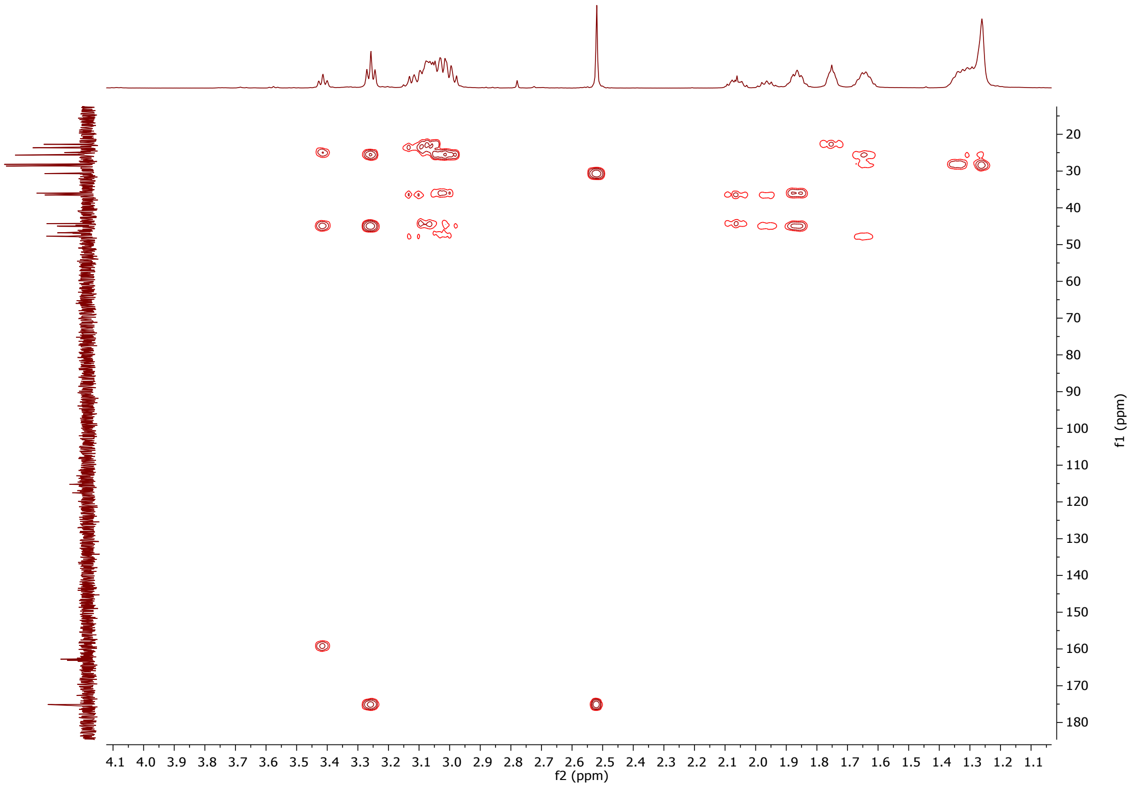

Fig. S15 ^{1}H-^{13}C HMBC NMR spectrum of compound **23** measured relative to the residual solvent peak (HOD) at 4.79 ppm in 99.7% D_2O at 25 °C.
